# Towards more precise automatic analysis: a systematic review of deep learning-based multi-organ segmentation

**DOI:** 10.1186/s12938-024-01238-8

**Published:** 2024-06-08

**Authors:** Xiaoyu Liu, Linhao Qu, Ziyue Xie, Jiayue Zhao, Yonghong Shi, Zhijian Song

**Affiliations:** 1 Digital Medical Research Center, School of Basic Medical Sciences, Fudan University, 138 Yixueyuan Road, Shanghai, 200032 People’s Republic of China; 2 Shanghai Key Laboratory of Medical Image Computing and Computer Assisted Intervention, Shanghai, 200032 China

**Keywords:** Abdomen multi-organ, Chest multi-organ, Deep learning, Head and neck multi-organ, Multi-organ segmentation

## Abstract

Accurate segmentation of multiple organs in the head, neck, chest, and abdomen from medical images is an essential step in computer-aided diagnosis, surgical navigation, and radiation therapy. In the past few years, with a data-driven feature extraction approach and end-to-end training, automatic deep learning-based multi-organ segmentation methods have far outperformed traditional methods and become a new research topic. This review systematically summarizes the latest research in this field. We searched Google Scholar for papers published from January 1, 2016 to December 31, 2023, using keywords “multi-organ segmentation” and “deep learning”, resulting in 327 papers. We followed the PRISMA guidelines for paper selection, and 195 studies were deemed to be within the scope of this review. We summarized the two main aspects involved in multi-organ segmentation: datasets and methods. Regarding datasets, we provided an overview of existing public datasets and conducted an in-depth analysis. Concerning methods, we categorized existing approaches into three major classes: fully supervised, weakly supervised and semi-supervised, based on whether they require complete label information. We summarized the achievements of these methods in terms of segmentation accuracy. In the discussion and conclusion section, we outlined and summarized the current trends in multi-organ segmentation.

## Introduction

Accurate segmentation of multiple organs in medical images is essential for various medical applications such as computer-aided diagnosis, surgical planning, navigation, and radiotherapy treatment [1, 2]. For instance, radiation therapy is a common treatment option for cancer patients, where tumor masses and high-risk microscopic areas are targeted [3]. However, radiation therapy can pose a significant risk to normal organs adjacent to the tumor, which are called organs at risk (OARs). Therefore, precise segmentation of both tumor and OARs contours is necessary to minimize the risk of radiation therapy [4, 5].

The early segmentation process relies heavily on manual labeling by physicians, which is labour-intense and time-consuming. For example, mapping 24 OARs in the head and neck region takes over 3 h, resulting in potential long waits for patients, especially in cases of patient overload [6]. Due to a shortage of experienced doctors, the mapping process becomes even more time-consuming, potentially delaying the patient's treatment process and missing the optimal treatment window [7]. Furthermore, the labeling results obtained by different physicians or hospitals exhibit significant variability [8–11]. Therefore, there is a pressing requirement for accurate and automated multi-organ segmentation methods in clinical practice.

Traditional methods [12–15] usually utilize manually extracted image features for image segmentation, such as the threshold method [16], graph cut method [17], and region growth method [18]. Limited by numerous manually extracted image features and the selection of non-robust thresholds or seeds, the segmentation results of these methods are usually unstable, and often yield only a rough segmentation result or only apply to specific organs. Knowledge-based methods leverage labeled datasets to automatically extract detailed anatomical information for various organs, reducing the need for manual feature extraction. This method can enhance the accuracy and robustness of multi-organ segmentation techniques, such as multi-atlas label fusion [19, 20] and statistical shape models [21, 22]. The method based on multi-atlas uses image alignment to align predefined structural contours to the image to be segmented. But this method typically includes multiple steps, therefore, the performance of this method may be influenced by various relevant factors involved in each step. Moreover, due to the use of fixed atlases, it is challenging to manage the anatomical variation of organs between patients. In addition, it is computationally intensive and takes a long time to complete an alignment task. The statistical shape model uses the positional relationships between different organs, and the shape of each organ in the statistical space as a constraint to regularize the segmentation results. However, the accuracy of this method is largely dependent on the reliability and extensibility of the shape model, and the model based on normal anatomical structures has very limited effect in the segmentation of irregular structures [23].

**Fig. 1:**
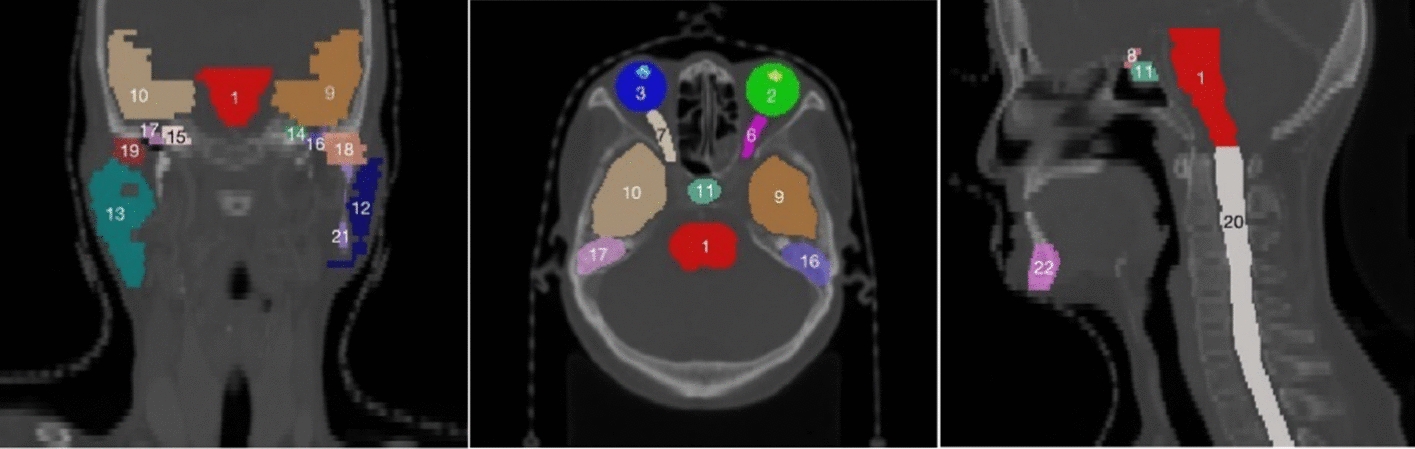
Schematic diagram of the organs of the head and neck, where the numbers are arranged in order: (1) brainstem, (2) left eye, (3) right eye, (4) left lens, (5) right lens, (6) left optic nerve, (7) right optic nerve, (8) Optical chiasm, (9) left temporal lobe, (10) right temporal lobe, (11) pituitary gland, (12) left parotid gland, (13) right parotid gland, (14) left temporal bone rock, (15) right temporal bone rock, (16) left temporal bone, (17) right temporal bone, (18) left mandibular condyle, (19) right mandibular condyle, (20) spinal cord, (21) left mandible, (22) right mandible. The segmentations and images are from the Automatic Radiotherapy Planning Challenge (StructSeg) in 2019 (https://structseg2019.grand-challenge.org/Dataset/)

**Fig. 2:**
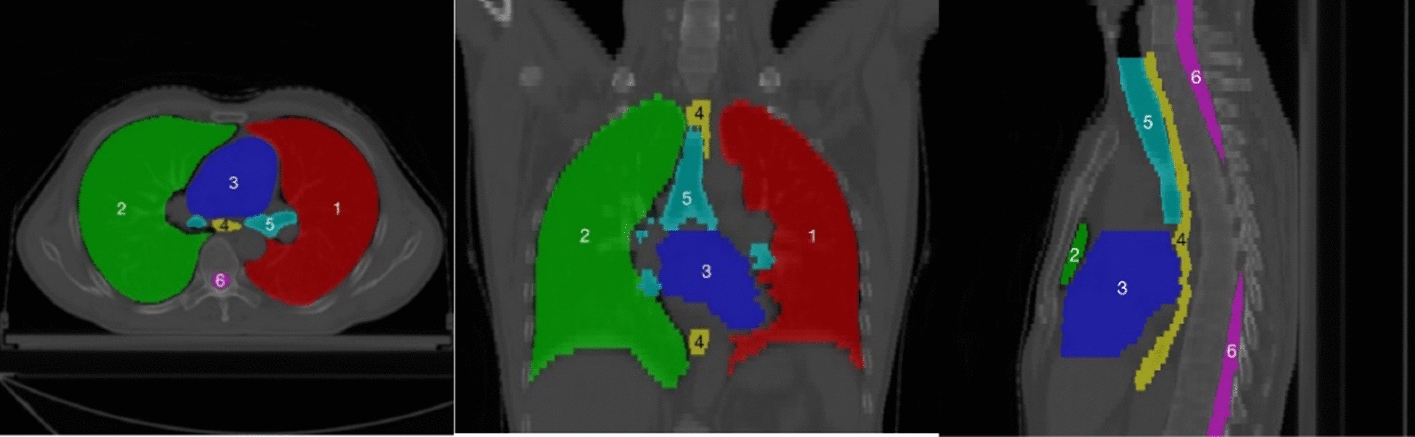
Schematic diagram of the thoracic organs, where the numbers are arranged in order: (1) left lung, (2) right lung, (3) heart, (4) esophagus, (5) trachea, and (6) spinal cord. The segmentations and images are from the Automatic Radiotherapy Planning Challenge (StructSeg) in 20191

**Fig. 3:**
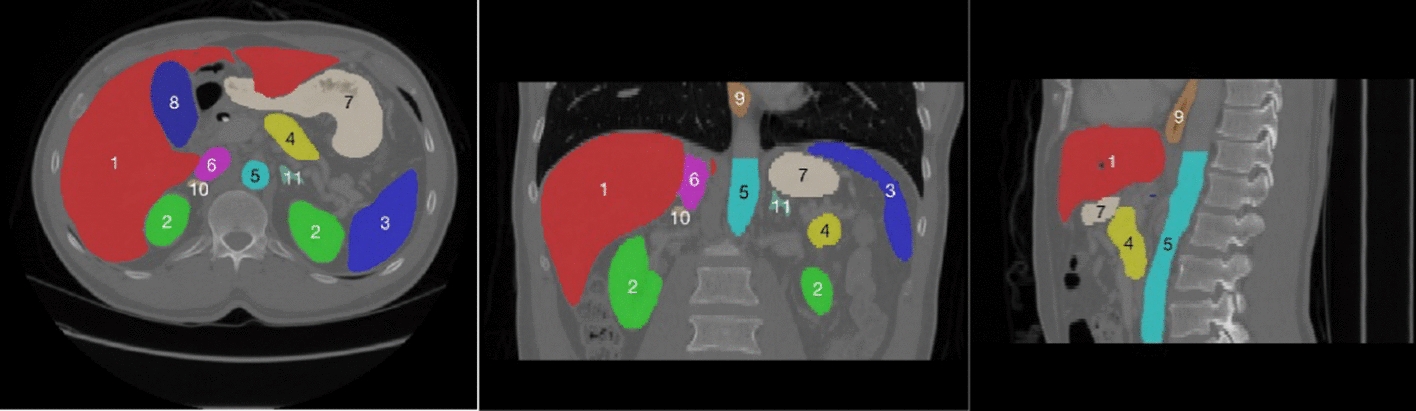
Schematic diagram of the abdominal organs, where the numbers are arranged in order: (1) liver, (2) kidney, (3) spleen, (4) pancreas, (5) aorta, (6) inferior vena cava, (7) stomach, (8) gallbladder, (9) esophagus, (10) right adrenal gland, (11) left adrenal gland, and (12) celiac artery. The segmentations and images are from the Multi-Atlas Labelling Beyond the Cranial Vault (BTCV) by MICCAI [34]

**Fig. 4:**
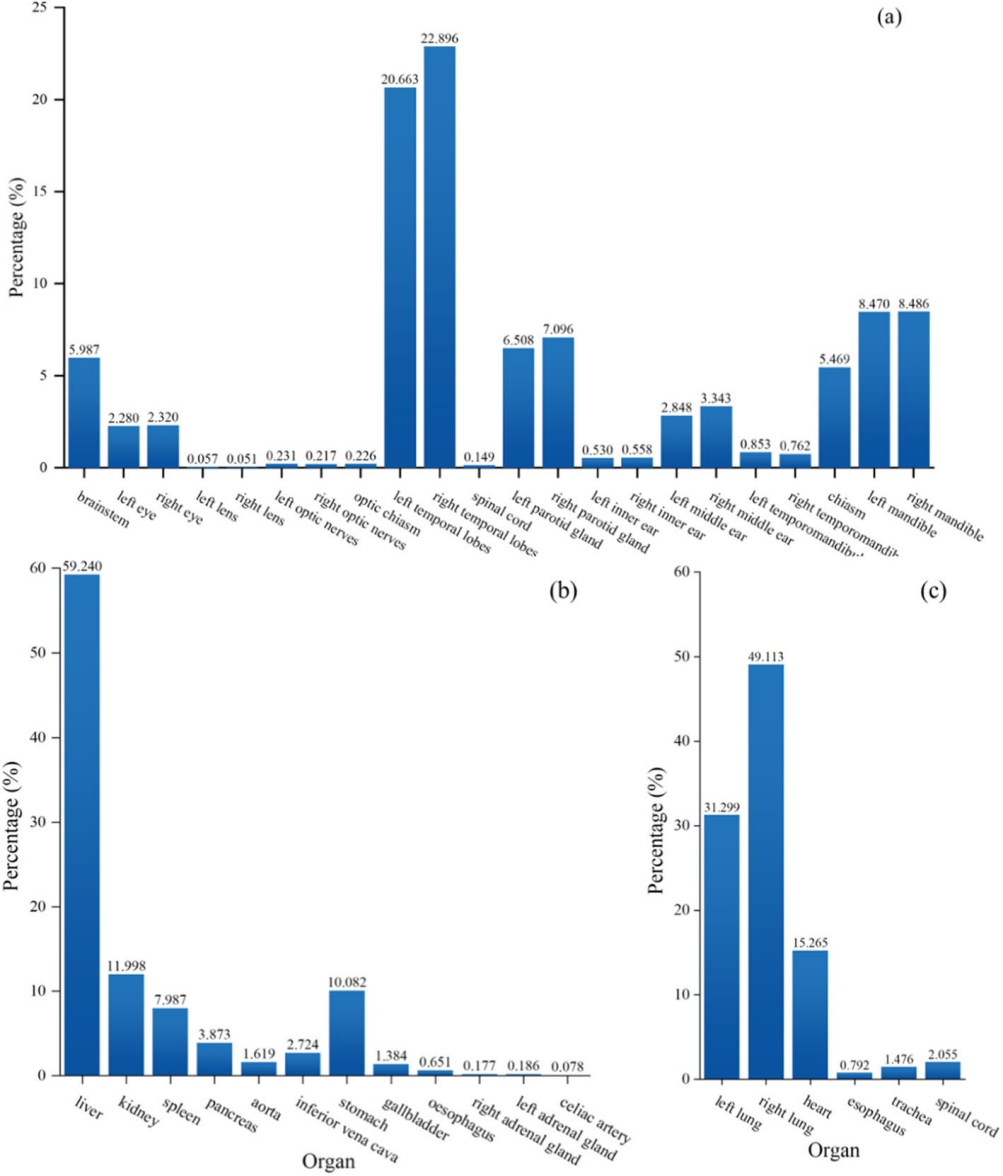
Illustration of the percentage of voxels in each organ of the head and neck (**a**), abdomen (**b**), and chest (**c**), respectively, which is calculated based on the BTCV data set [34]

**Fig. 5:**
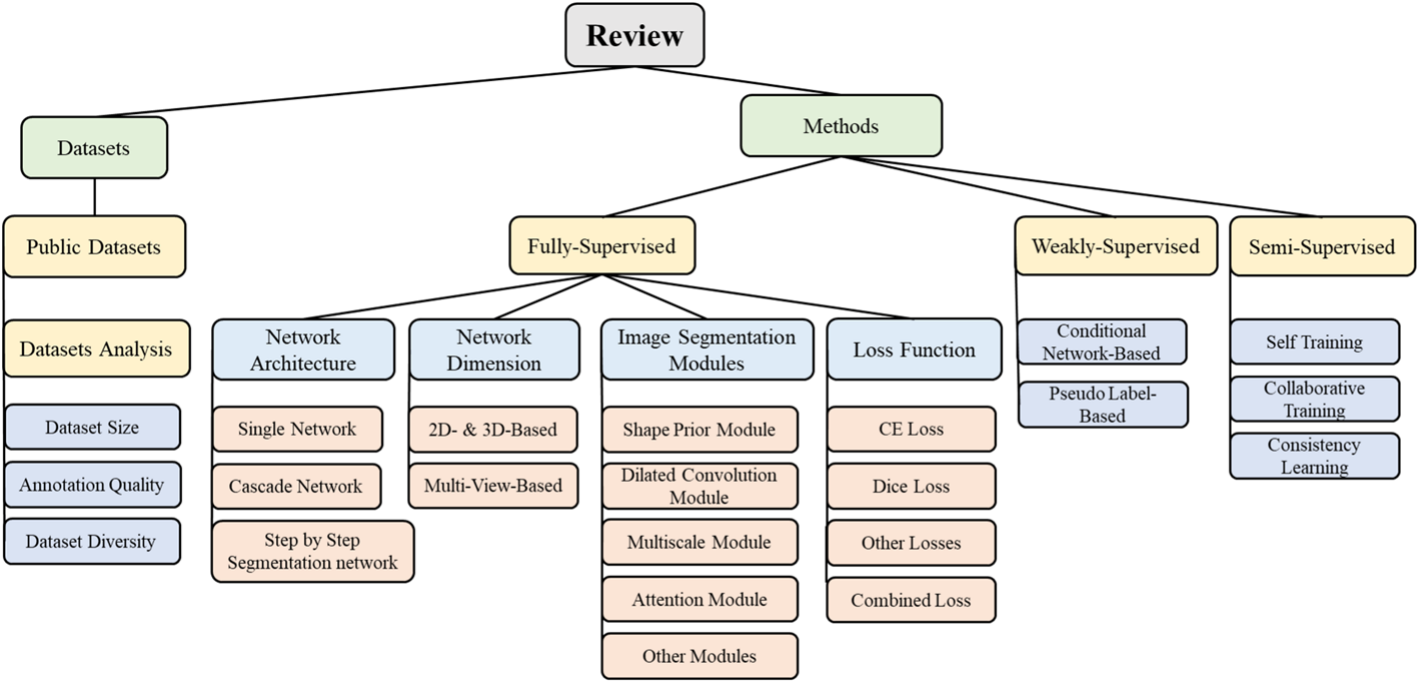
Framework diagram of the overview

Compared to traditional methods that require manual feature extraction, deep learning can automatically learn the parameters of the model from a large number of data samples, enabling the model to learn complex features and patterns from the data. Recently, deep learning-based methods have gained considerable attention in several image processing applications such as image classification [24], object detection [25], image segmentation [26, 27], image fusion [28], image registration [29] due to their ability to extract features automatically. Methods based on deep learning have become a mainstream in the field of medical image processing. However, there are still several major challenges in multi-organ segmentation tasks. Firstly, there are significant variations in organ sizes, as illustrated by the head and neck in Fig. 1, the chest in Fig. 2, the abdomen in Fig. 3, and the organ size statistics in Fig. 4. Such size imbalances can lead to poor segmentation performance of the trained network for small organs. Secondly, the inherent noise and low contrast in CT images often result in ambiguous boundaries between different organs or tissue regions, thereby reducing the accuracy of organ boundary segmentation achieved by segmentation networks. Finally, due to safety and ethical concerns, many hospitals do not disclose their datasets, as a result, datasets used to train multiple organ segmentation models are very limited, and many segmentation methods are trained and validated on private datasets, making it difficult to compare with other methods. Consequently, there is an increasing demand for the development of multi-organ segmentation techniques that can accurately segment organs of different sizes, as shown in Fig. 5.

Recently, only a few comprehensive reviews have provided detailed summaries of existing multi-organ segmentation methods. For example, Fu et al*.* [30] summarized literature of deep learning-based multi-organ segmentation methods up to 2020, providing a comprehensive overview of developments in this field; Vrtovec et al*.* [31] systematically analyzed 78 papers published between 2008 and 2020 on the automatic segmentation of OARs in the head and neck. However, these reviews encounter certain issues. Firstly, with the rapid development of technology, many novel methods such as transformer architecture [32], foundation models [33] have emerged for addressing multi-organ segmentation, and more public datasets have also been introduced. However, these reviews only encompassed literature up to 2020; secondly, they categorized methods solely based on network design, without categorizing and summarizing specific solutions unique to the challenges of multi-organ segmentation; thirdly, the majority of these reviews primarily covered fully supervised methods and did not provide a summary of papers related to weakly supervised and semi-supervised; lastly, they did not provide a comprehensive summary of the segmentation accuracy for each organ, making it difficult for readers to assess the current segmentation precision for each organ and knew which organs have reached a mature stage of segmentation and which organs still pose challenges.

In this review, we have summarized around the datasets and methods used in multi-organ segmentation. Concerning datasets, we have provided an overview of existing publicly available datasets for multi-organ segmentation and conducted an analysis of these datasets. In terms of methods, we categorized them into fully supervised, weakly supervised, and semi-supervised based on whether complete pixel-level annotations are required. Within the fully supervised methods, we organized the methods according to the network architectures used, input image dimensions, segmentation modules specifically designed for multi-organ segmentation, and the loss functions employed. For weakly supervised and semi-supervised methods, we summarized the latest papers in each subcategory. Detailed information on the datasets and network architectures used in each paper, along with the segmentation accuracy achieved for each organ, has been provided to enable readers to quickly understand the current segmentation accuracy of each organ on the respective datasets. In the discussion section, we have summarized the existing methods in this field and, in conjunction with the latest technologies, discussed future trends in the field of multi-organ segmentation.

The structure of this review is as follows. The first section elaborates on the mathematical definition of multi-organ segmentation and the corresponding evaluation metrics. The second section describes how we conducted literature research and screening based on PRISMA [35]. The third section presents the literature analysis we retrieved, categorized into two main sections: data and methods. In the data section, we summarize existing public datasets and conduct analysis. In the methods section, we divide into three categories: supervised methods, weakly and semi-supervised methods. In the fourth section, we discuss existing methods and their future prospects, while in the fifth section, we summarize the entire paper.

### Definition and evaluation metrics

Let ${\varvec{X}}$ represent the union of input images, ${\varvec{G}}$ represent the union of ground truth labels, ${\varvec{P}}$ represent the union of predicted labels, ***f*** represents the neural network, and ${\varvec{\theta}}$ represents its parameters, where ${\varvec{P}}={\varvec{f}}({\varvec{X}};\boldsymbol{ }{\varvec{\theta}})$.

**Fig. 6:**
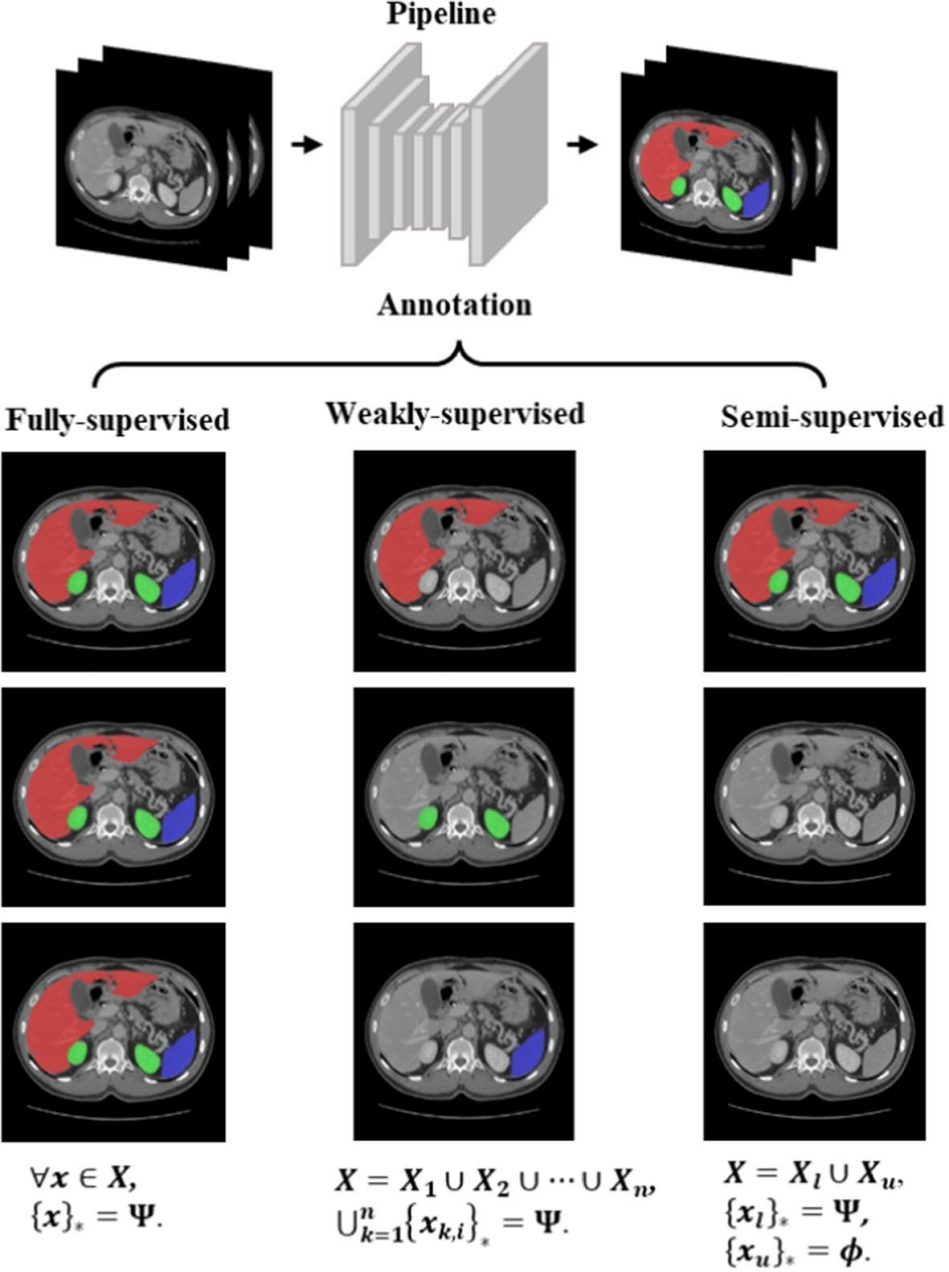
General overview of the learning paradigms reviewed in this paper. (The images presented in this figure are sourced from the MICCAI Multi-Atlas Labelling Beyond the Cranial Vault (BTCV) data set [34].)

Given a multi-organ segmentation task, ${\varvec{\Psi}}$ represents the class set of organs to be segmented. ${\left\{{\varvec{x}}\right\}}_{\boldsymbol{*}}$ represents the set of organs annotated in ${\varvec{x}}$. According to the available annotations, multi-organ segmentation can be implemented according to three learning paradigms, as shown in Fig. 6: fully supervised learning, weakly supervised learning, and semi-supervised learning. Fully supervised learning means that the labels of all organ are given, which indicates that $\forall {\varvec{x}}\in {\varvec{X}},\boldsymbol{ }{\left\{{\varvec{x}}\right\}}_{\boldsymbol{*}}={\varvec{\Psi}}$. Weakly supervised learning often means that the data come from ${\varvec{n}}$ different datasets. However, each dataset provides the annotations of one or more organs but not all organs, which means that ${\varvec{X}}={{\varvec{X}}}_{1}\cup {{\varvec{X}}}_{2}\cup \cdots \cup {{\varvec{X}}}_{n},\boldsymbol{ }\boldsymbol{ }\forall \boldsymbol{ }{{\varvec{x}}}_{k,i}\in {{\varvec{X}}}_{k}, k=\mathrm{1,2},\dots n,\boldsymbol{ }\boldsymbol{ }{\left\{{{\varvec{x}}}_{k,i}\right\}}_{\boldsymbol{*}}\subseteq{\varvec{\Psi}}$,$\bigcup_{k=1}^{n}{\left\{{{\varvec{x}}}_{k,i}\right\}}_{\boldsymbol{*}}={\varvec{\Psi}}$. Here, ${{\varvec{x}}}_{{\varvec{k}},{\varvec{i}}}$ denotes the *i*th image in ${{\varvec{X}}}_{{\varvec{k}}}$. Semi-supervised learning indicate that some of the training datasets are fully labeled and others are unlabelled, ${\varvec{X}}={{\varvec{X}}}_{{\varvec{l}}}\cup {{\varvec{X}}}_{{\varvec{u}}}$. ${{\varvec{X}}}_{{\varvec{l}}}$ represents the fully labeled dataset, ${{\varvec{X}}}_{{\varvec{u}}}$ represents the unlabelled dataset, which indicates that $\forall {{\varvec{x}}}_{{\varvec{l}}}\in {{\varvec{X}}}_{{\varvec{l}}},\boldsymbol{ }{\left\{{{\varvec{x}}}_{{\varvec{l}}}\right\}}_{\boldsymbol{*}}={\varvec{\Psi}}$ and $\forall {{\varvec{x}}}_{{\varvec{u}}}\in {{\varvec{X}}}_{{\varvec{u}}},\boldsymbol{ }{\left\{{{\varvec{x}}}_{{\varvec{u}}}\right\}}_{\boldsymbol{*}}={\varvec{\phi}}$**,** which represents the empty set**,** and the size of ${{\varvec{X}}}_{{\varvec{l}}}$ is far less than the one of ${{\varvec{X}}}_{{\varvec{u}}}$.

The performance of the segmentation methods is typically evaluated using metrics such as the *Dice Similarity Coefficient* (*DSC*), *95% Hausdorff Distance* (*HD95*) and *Mean Surface Distance (MSD)*. *DSC* is a measure of the volume overlap between the predicted outputs and ground truth, *HD95* and *MSD* are measures of the surface distance between them:

1 $$DSC=\frac{2\times |{P}^{c}\cap {G}^{c}|}{\left|{P}^{c}\right|+|{G}^{c}|},$$

2 $$HD95={max}_{95\mathrm{\%}}\left[d\left({P}_{s}^{c},{G}_{s}^{c}\right),d\left({G}_{s}^{c},{P}_{s}^{c}\right)\right],$$

3 $$MSD=\frac{1}{\left|{P}_{s}^{c}\right|+\left|{G}_{s}^{c}\right|}\left(\sum_{{p}_{s}^{c}\in {P}_{s}^{c}}d\left({p}_{s}^{c},{G}_{s}^{c}\right)+\sum_{{g}_{s}^{c}\in {G}_{s}^{c}}d\left({g}_{s}^{c},{P}_{s}^{c}\right)\right),$$

where ${P}^{c}$ and ${G}^{c}$ represent the set of predicted pixels and the set of real pixels of the $c$ class organ, respectively; ${P}_{s}^{c}$ and ${G}_{s}^{c}$ represent the set of predicted pixels and the set of real pixels of the surface of the $c$ class organ, respectively; and $d\left({p}_{s}^{c},{G}_{s}^{c}\right)={min}_{{g}_{s}^{c}\in {G}_{s}^{c}}{||{p}_{s}^{c}-{g}_{s}^{c}||}_{2}$ represents the minimal distance from point ${p}_{s}^{c}$ to surface ${G}_{s}^{c}$. The review reports various methods based on ***DSC*** values.

### Search protocol

This paper adopts the method proposed by the PRISMA guidelines [35] to determine the articles included in the analysis. The articles were primarily obtained through Google Scholar. Using the keywords “multi-organ segmentation” and “deep learning”, the search covered the period from January 1, 2016, to December 31, 2023, resulting in a total of 327 articles. We focused on highly cited articles, including those published in top conferences (such as NeurIPS, CVPR, ICCV, ECCV, AAAI, MICCAI, etc.) and top journals (such as TPAMI, TMI, MIA, etc.). Two researchers independently reviewed these articles to determine their eligibility. Among them, 67 articles did not meet the inclusion criteria based on the title and abstract, and 45 complete manuscripts were evaluated separately. In the end, we included 195 studies for analysis.

## Result

### Datasets

#### Public datasets

To obtain high-quality datasets for multi-organ segmentation, numerous research teams have collaborated with medical organizations. A summary of commonly used datasets for validating multi-organ segmentation methods in the head and neck, thorax, and abdomen regions can be found in Table 1, with references in [34, 36–49]. The table also reveals that the amount of annotated data available for deep learning studies remains insufficient.

**Tabel 1:** Frequently used datasets for multi-organ segmentation

Year	Download link	Dataset	Modality	Region	Targets	Scans	Annotated organs or tumors
2017	https://www.imagenglab.com/newsite/pddca/	Public Domain Database for Computational Anatomy (PDDCA) [36]	CT	Head and neck	9	48	Brainstem, Mandible, Chiasm, Left_optic_nerves, Right_optic_nerves, Left_parotid_glands, Right_parotid_glands, Left_submandibular_glands, Right_submandibular_glands
2017	https://zenodo.org/record/7442914#.ZBtfBHbMJaQ	The Head and Neck Organ-at-Risk CT & MR Segmentation Challenge(HaN-Seg)[46]	CT&MR	Head and neck	30	42	Carotid_artery(2), Arytenoids, Mandible, Brainstem, Buccal_mucosa, Cavity_oral, Cochlea(2), Cricopharyngeus, Esophagus, Eye(4), Glnd_Lacrimal(2), Glnd_Submand(2), Glnd_Thyroid, Glottis, Larynx_SG, Lips, OpticChiasm, Opticnrv(2), Parotid, Pituitary, SpinalCord
2019	https://structseg2019.grand-challenge.org/Dataset/	Automatic Structure Segmentation for Radiotherapy Planning Challenge 2019 (StructSeg)	CT	Head and neck	22	50	Left_eye, Right Eye, Left Lens, Right Lens, Left Optical Nerve, Right Optical Nerve, Chiasm, Pituitary, Brainstem, Left Temporal Lobes, Right Temporal Lobes, Spinal Cord, Left Parotid Gland, Right Parotid Gland, Left Inner Ear, Right Inner Ear, Left Middle Ear, Right Middle Ear, Left Temporomandibular Joint, Right Temporomandibular Joint, Left Mandible, Right Mandible
2020	https://github.com/ababier/open-kbp	OpenKBP: The open-access knowledge-based planning grand challenge and dataset [41]	CT	Head and neck	7	340	Brainstem, Spinal Cord, Right Parotid, Left Parotid, Larynx, Esophagus, Mandible
2023	https://segrap2023.grand-challenge.org/dataset/	Segmentation of Organs-at-Risk and Gross Tumor Volume of NPC for Radiotherapy Planning(SegRap 2023)[47]	CT	Head and neck	45	200	Brain, Brainstem, Chiasm, Temporallobe, Temporallobe_Hippocampus_OverLap(2), Hippocampus, Eye(2), Lens(2), OpticNerve(2), MiddleEar(2), IAC(2), MiddleEar_TympanicCavity_OverLap(2), TympanicCavity(2), MiddleEar_VestibulSemi_OverLap(2), VestibulSemi(2), Cochlea(2), MiddleEar_ETbone_OverLap(2), ETbone(2), Pituitary, OralCavity, Mandible(2), Submandibular(2), Parotid(2), Mastoid(2), TMjoint(2), SpinalCord, Esophagus, Larynx, Larynx_Glottic, Larynx_Supraglot, Larynx_PharynxConst_OverLap, PharynxConst, Thyroid, Trachea
2017	https://www.cancerimagingarchive.net/	Thoracic Auto-segmentation Challenge (AAPM) [37]	CT	Thorax	5	60	Left Lung, Right Lung, Heart, Esophagus, Spinal Cord
2019	https://competitions.codalab.org/competitions/21145	SegTHOR Challenge: Segmentation of Thoracic Organs at Risk in CT Images (SegTHOR) [45]	CT	Thorax	5	60	Trachea, Heart, Esophagus, Aorta
2019	https://structseg2019.grand-challenge.org/Dataset/	Automatic Structure Segmentation for Radiotherapy Planning Challenge (StructSeg)	CT	Thorax	6	50	Left Lung, Right Lung, Spinal Cord, Esophagus, Heart, Trachea
2010	https://cloud.ircad.fr/index.php/s/JN3z7EynBiwYyjy/download	3D-IRCADb	CT	Abdomen	22	40	skin, kidney(2), lung(2), portal_vein_and_splenic_vein, artery, spleen, venous_system, liver, bone, liver_cyst, liver_tumor, venacava, kidney, biliary_system, metal, stones, gallbladder, pancreas, stomach, colon, heart, aorta, hyperplasle, lymph_nodes, surrenalgland_left, tumor, metastasectomy, lung, bladder, surrenalgland, small_intestin, uterus, esophagus, surrenalgland_right, duodenum, sigmoid, leftsurre_tumor, rightsurre_tumor
2015	https://www.synapse.org/#!Synapse:syn3193805/wiki/89480	MICCAI Multi-Atlas Labelling Beyond the Cranial Vault (BTCV) [34]	CT	Abdomen	13	50	Spleen, Right Kidney, Left Kidney, Gallbladder, Esophagus, Liver, Stomach, Aorta, Inferior Vena Cava, Portal and Splenic Veins, Pancreas, Right Adrenal Gland, Left Adrenal Gland
2019	https://chaos.grand-challenge.org/Combined_Healthy_Abdominal_Organ_Segmentation/	Combined (CT-MR) Healthy Abdominal Organ Segmentation (CHAOS) [38]	CT&MR	Abdomen	4	40	Spleen, Right Kidney, Left Kidney, Liver
2021	https://github.com/JunMa11/AbdomenCT-1K	Abdomenct-1 k [39]	CT	Abdomen	5	1112	Spleen, Right Kidney, Left Kidney, Liver, Pancreas
2019	https://drive.google.com/drive/folders/1HqEgzS8BV2c7xYNrZdEAnrHk7osJJ--2	Medical Segmentation Decathlon (MSD) [41]	CT	Abdomen	13	947	Spleen, Liver and Liver Tumor, Lung Tumor, Colon Tumor, Pancreas and Pancreas Tumor, Hepatic Vessel and Hepatic Vessel Tumor
2022	https://amos22.grand-challenge.org/	A Large-Scale Abdominal Multi-Organ Benchmark for Versatile Medical Image Segmentation (AMOS) [42]	CT	Abdomen	15	500	Spleen, Right Kidney, Left Kidney, Gallbladder, Esophagus, Liver, Stomach, Aorta, Inferior Vena Cava, Pancreas, Right Adrenal Gland, Left Adrenal Gland, Duodenum, Bladder, Prostate/Uterus
			MR			100	
2022	https://github.com/HiLab-git/WORD	Whole Abdominal Organ Dataset (WORD) [43]	CT	Abdomen	16	150	Spleen, Right Kidney, Left Kidney, Gallbladder, Esophagus, Liver, Stomach, Pancreas, Right Adrenal Gland, Duodenum, Colon, Intestine, Rectum, Bladder, Left Femur, Right Femur
2023	https://codalab.lisn.upsaclay.fr/competitions/12239#learn_the_details-dataset	FLARE 2023 [48]	CT	Abdomen	14	4500	Liver, Right kidney, Spleen, Pancreas, Aorta, Inferior vena cava, Right adrenal gland, Left adrenal gland, Gallbladder, Esophagus, Stomach, Duodenum, Left Kidney, Tumor
2020	https://wiki.cancerimagingarchive.net/pages/viewpage.action?pageId=61080890	CT volumes with multiple organ segmentations (CT-ORG) [44]	CT	Total	6	140	Liver, Bladder, Lungs, Kidneys, Bone, Brain
2023	https://zenodo.org/records/10047292	TotalSegmentator[49]	CT	Total	117	1228	Spleen, Kidney(2), Gallbladder, Liver, Stomach, Pancreas, Adrenal_gland (2), Lung_lobe(5), Esophagus, Trachea, Thyroid_gland, Small_bowel, Duodenum, Colon, Bladder, Prostate, Kidney_cyst(2), Sacrum, Vertebrae(25), Heart, Aorta, Pulmonay_vein, Brachiocephalic_trunk, Susclavian_artery(2), Common_carotid_artery(2), Brachiocephalic_vein(2), Atrial_appendage_left, Superior_vena_cava, Inferior_vena_cava, Portal_vein_and_splenic_vein, Iliac_artery(2), Iliac_vena(2), Humerus(2), Scapula(2), Clavicula(2), Femur(2), Hip(2), Spinal_cord, Gluteus(6), Autochthon(2), Iliopsoas(2), Brain, Skull, Rib(24), Sternum, Costal_cartilages

#### Datasets analysis

Data play a crucial role in improving model performance. In certain cases, such as lung segmentation, the key issue has shifted from algorithm complexity to dataset quality. Accurate lung segmentation does not necessarily require complex techniques [50]. Even with simple network architectures, superior results can be achieved with more extensive and heterogeneous private data. The lack of diversity in training data is considered one of the primary obstacles to building robust segmentation models.

Therefore, acquiring large-scale, high-quality, and diverse multi-organ segmentation datasets has become an important direction in current research. Due to the difficulty of annotating medical images, existing publicly available datasets are limited in number and only annotate some organs. Additionally, due to the privacy of medical data, many hospitals cannot openly share their data for training purposes. For the former issue, techniques such as semi-supervised and weakly supervised learning can be utilized to make full use of unlabeled and partially labeled data. Alternatively, human-in-the-loop [51] techniques can combine human knowledge and experience with machine learning to select samples with the highest annotation value for training. For the latter issue, federated learning [52] techniques can be applied to achieve joint training of data from various hospitals while protecting data privacy, thus fully utilizing the diversity of the data.

#### Dataset size

Incorporating unannotated data into training or integration; existing partially labeled data can be fully utilized to enhance model performance, as detailed in Section of Weakly and semi-supervised methods.

#### Annotation quality

Human-in-the-loop integration of human knowledge and experience minimizes the cost of training accurate predictive models [51]. By closely collaborating, humans and machines leverage each other’s primary strengths to maximize efficiency. Human-in-the-loop primarily consists of two categories: active learning [53] and interactive segmentation [54]. Active learning selects the next batch of annotated samples through algorithms to maximize model performance, presenting an economically effective method for expanding training datasets. Another category, interactive segmentation, expedites the annotation process by allowing expert annotators to interactively correct initial segmentation masks generated by the model.

Wang et al*.*[55] comprehensively reviewed core methods of deep active learning, including informative assessment, sampling strategies, integration with other techniques such as semi-supervised and self-supervised learning, and customized active learning works specifically for medical image analysis. Recently, Qu et al*.*[56] proposed a novel and systematically effective active learning-based organ segmentation and labeling method. They annotated spleen, liver, kidney, stomach, gallbladder, pancreas, aorta, and inferior vena cava in 8,448 CT volumes. The proposed active learning process generated an attention map, highlighting areas that radiologists need to modify, reducing annotation time from 30.8 years to 3 weeks and accelerating the annotation process by 533 times.

Interactive segmentation in medical imaging typically involves a sequential interactive process, where medical professionals iteratively improve annotation results until the desired level of accuracy is achieved [57]. In recent years, many deep learning-based interactive segmentation methods have been proposed. Recent advancements in natural image segmentation have witnessed the emergence of segmentation-agnostic models like the Segmentation Anytime Model (SAM) [58, 59], demonstrating remarkable versatility and performance in various segmentation tasks. Various large models for medical interactive segmentation have also been proposed, providing powerful tools for generating more high-quality annotated datasets.

#### Dataset diversity

One significant reason for the limited availability of data for multi-organ segmentation is the issue of data privacy. Many institutions are unable to share their data for training due to privacy concerns. The emergence of federated learning addresses this problem precisely. Federated learning is a distributed learning approach in machine learning aimed at training models across multiple devices or data sources without centralizing the dataset in a single location. In federated learning, model training occurs on local devices, and then locally updated model parameters are sent to a central server, where they are aggregated to update the global model [52]. This distributed learning approach helps protect user privacy because data do not need to leave devices for model training.

In federated learning, the heterogeneity of statistical data is a crucial research issue. FedAvg is one of the pioneering works to address this issue, using weighted averaging of local weights based on local training scale and has been widely recognized as a baseline for federated learning [60]. Recently, several federated learning algorithms have been proposed for medical image segmentation tasks. For example, FedSM [61] employs a model selector to determine the model or data distribution closest to any testing data. Studies [62] have shown that architectures based on self-attention exhibit stronger robustness to distribution shifts and can converge to better optimal states on heterogeneous data.

Federated learning enables data from multiple sites to participate in training simultaneously without requiring hospitals to disclose their data, thereby enhancing dataset diversity and training more robust segmentation models.

### Methods

#### Fully supervised methods

The fully supervised methods require complete annotation of all organs involved in the multi-organ segmentation task. The existing methods can be analyzed from four parts: network architecture, network dimension, image segmentation modules, and network loss function. The network architecture is further divided into single network, cascade network and step-by-step segmentation networks; while network dimension categorizes methods based on the image dimension used (2D, 3D, or multi-view); image segmentation modules refer to modules that are frequently used in multi-organ segmentation to improve segmentation performance, and network loss function summarizes the innovative use of common loss functions for multi-organ segmentation.

#### Network architecture

**Fig. 7:**
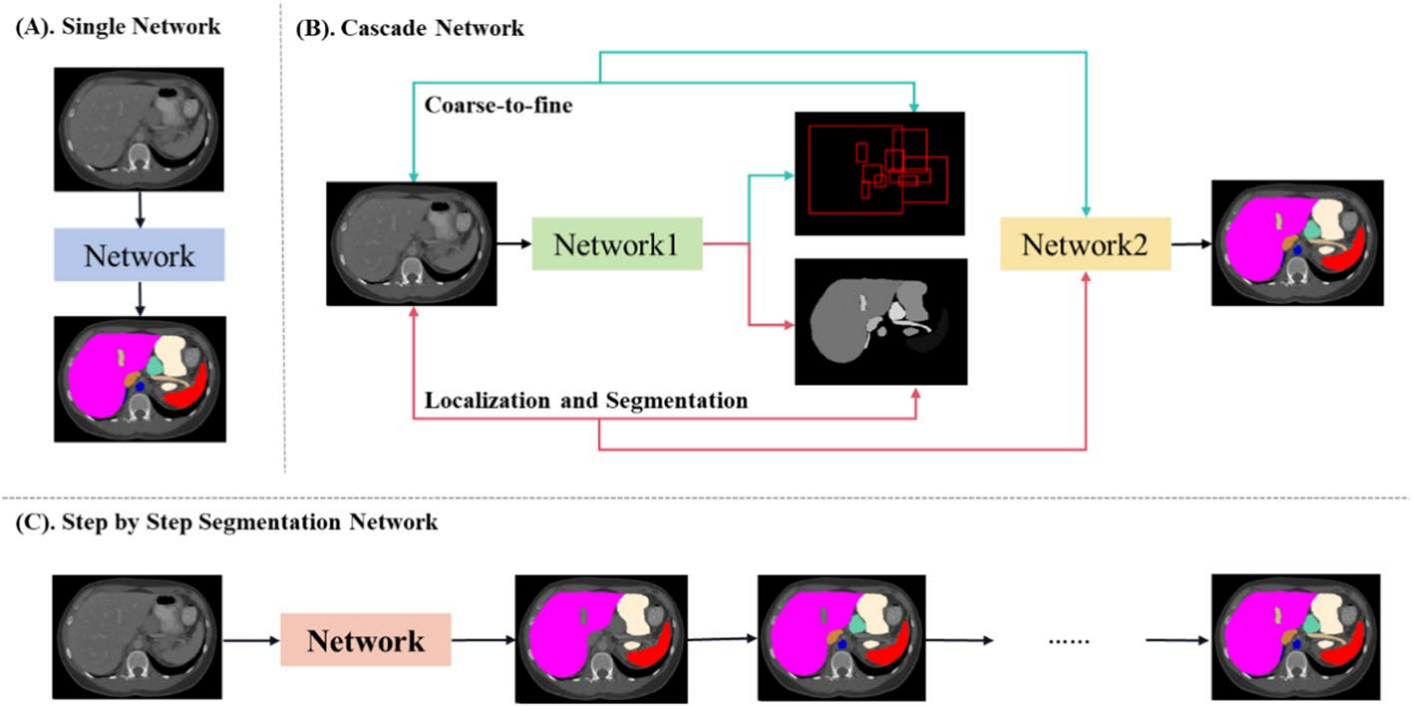
Three architecture of multi-organ segmentation network

Multi-organ segmentation methods can be categorized based on their network architecture, which can be divided into three types: single network, cascade network, and step-by-step segmentation network, which is shown in Fig. 7. Tables 2, 3, 4 summarize the literature related to methods for the segmentation of multi-organ in the head and neck, abdomen and chest based on DSC metrics. Since there are so many organs in the head and neck as well as the abdomen, this paper mainly reports on 9 organs in the head and neck and 7 organs in the abdomen. Tables 5, 6 summarize the DSC values of other organs.

**Table 2:** DSC-based summary of the paper about head and neck

Year	Refs.	Backbone	Datasets	Scans	Targets	Brainstem	Mandible	Parotid gland		Submandibular Gland		Optic nerve		Chiasm
								Left	Right	Left	Right	Left	Right	
2017	Ibragimov and Xing [67]	2.5D CNN	Private (CT)	50	13	–	0.895	0.766	0.779	0.697	0.730	0.639	0.645	0.374
2016	Fritscher et al*.*[68]	2.5D CNN	HNC (CT) [36]	30	3	–	–	0.810	–	0.650	–	–	–	0.520
2019	Zhu et al*.* [75]	3D U-Net	Private (CT)	261	9	0.867	0.925	0.881	0.874	0.814	0.813	0.721	0.706	0.532
2019	Van Rooij et al*.* [76]	3D U-Net	Private (CT)	157	11	0.640	–	0.830	0.830	0.820	0.810	–	–	–
2019	Tong et al*.* [100]	3D GAN	PDDCA (CT) [36]	48	9	0.867	0.939	0.855	0.858	0.807	0.819	0.664	0.699	0.592
2019	Tong et al*.* [100]	3D GAN	Private (MRI)	25	9	0.916	0.816	0.865	0.825	–	–	0.717	0.693	0.589
2020	Gou et al*.* [77]	3D U-Net	HNC (CT) [36]	48	9	0.880	0.940	0.870	0.860	0.780	0.810	0.720	0.710	0.610
2020	Liu et al*.* [82]	3D U-Net	Private (MRI & CT)	45	19	0.880	0.890	0.890	0.880	–	–	0.720	0.720	0.760
2020	Liu et al*.* [82]	3D U-Net	HNC (CT) [36]	48	9	0.910	0.960	0.880	0.880	0.860	0.850	0.780	0.780	0.730
2021	Chen et al*.* [164]	2.5D U-Net	Private (CT)	307	24	–	–	–	–	–	–	0.711	0.712	0.598
2021	Chen et al*.* [164]	2.5D U-Net	HNC (CT) [36]	48	9	0.872	0.922	0.867	0.858	0.821	0.821	0.750	0.741	0.663
2020	Liu et al*.* [189]	2D U-Net	StructSeg (CT)	50	22	0.864	0.906	0.802	0.826	–	–	0.770	0.647	0.712
2021	Cros et al*.* [83]	3D U-Net	Private (CT)	200	12	–	0.900	0.760	0.760	0.740	–	–	–	–
2021	Lei et al*.* [211]	2.5D U-Net	StructSeg (CT)	50	22	0.897	0.914	0.857	0.873	–	–	0.680	0.663	0.566
2021	Lei et al*.* [211]	2.5D U-Net	Hybrid HAN (CT)	165	7	0.874	0.900	0.847	0.846	–	–	0.624	0.621	0.290
2021	Zhang et al*.*[78]	2D U-Net	HNC (CT) [36]	48	9	0.840	0.900	0.820	0.830	0.820	0.810	0.670	0.710	0.660
2022	Srivastava et al*.* [186]	3D U-Net	OpenKBP (CT) [40]	188	5	0.803	0.883	0.799	0.773	–	–	–	–	–
2022	Podobnik et al*.*[95]	2D nnU-net	Private (CT&MR)	56	31	0.836	0.898	0.817	0.765	0.716	0.670	0.572	0.604	0.387
2022	Kan et al*.* [125]	3D Transformer and U-Net	Private (CT)	94	18	0.871	0.925	0.821	0.844	–	–	0.717	0.679	0.328
2022	Jiang et al*.* [84]	2D U-Net	PDDCA (CT) [36]	16	6	0.920	0.950	0.880	0.880	0.820	0.830	–	–	–
2022	Francis et al*.*[85]	3D U-Net	Private (CT)	232	7	0.890	0.932	0.852	0.870	–	–	0.744	0.764	0.635
			HNC (CT) [36]	48	7	0.862	0.940	0.885	0.885	–	–	0.728	0.723	0.620
2022	Isler et al*.* [181]	2D U-Net	HNC (CT) [36]	48	6	0.830	-	0.790	0.760	–	–	0.580	0.540	0.520
			OpenKBP (CT) [40]	188	5	0.800	0.860	0.750	0.760	–	–	–	–	–
2019	Tappeiner et al. [131]	3D CNN and 3D CNN	HNC (CT) [36]	40	7	0.820	0.910	0.800	0.810	–	–	0.640	0.630	0.420
2020	Pu et al*.* [169]	2.5D U-Net and 3D U-Net	HNC (CT) [36]	48	9	0.880	0.940	0.860	0.865	0.788	0.802	0.743	0.768	0.612
2021	Ma et al*.* [154]	3D U-Net and 3D U-Net	HNC (CT) [36]	48	9	0.879	0.945	0.892	0.884	0.829	0.815	0.753	0.747	0.659
2022	Fang et al*.* [133]	2D FCN and 3D U-Net	HNC (CT) [36]	32	9	0.849	0.924	0.842	0.849	0.734	0.782	0.676	0.684	0.547
			Private (CT)	56	14	0.863	0.905	0.582	0.687	0.668	0.575	–	–	–
2019	Wang et al*.* [142]	3D U-Net and 3D U-Net	HNC (CT) [36]	48	9	0.875	0.930	0.864	0.848	0.758	0.733	0.737	0.736	0.451
2018	Men et al*.* [143]	3D U-Net and 3D U-Net	TCIA (CT)	100	7	0.900	0.920	0.860	0.860	–	–	–	–	–
2019	Tang et al*.* [144]	3D U-Net and 3D U-Net	Private (CT)	215	28	0.863	0.931	0.849	0.849	0.807	0.825	0.757	0.761	0.642
2019	Tang et al*.* [144]	3D U-Net and 3D U-Net	PDDCA (CT) [36]	48	9	0.875	0.950	0.887	0.875	0.823	0.815	0.748	0.723	0.615
2019	Yang et al*.* [145]	3D CNN and 2D U-Net	Private (CT)	88	17	0.831	0.875	0.807	0.811	–	–	0.638	0.675	–
2019	Liang et al*.* [146]	2D CNN and 2D CNN	Private (CT)	185	18	0.896	Left: 0.914; Right: 0.912	0.852	0.850	–	–	0.661	0.717	–
2019	Gao et al*.* [147]	3D CNN and 3D CNN	Private (CT)	50	18	0.858	–	0.772	0.800	–	–	0.639	0.617	0.638
2019	Gao et al*.* [147]	3D CNN and 3D CNN	HNC (CT) [36]	48	9	0.875	0.935	0.863	0.879	0.798	0.801	0.735	0.744	0.596
2020	Liang et al*.* [148]	2.5D CNN and 2.5D CNN	HNC (CT) [36]	48	9	0.923	0.941	0.876		0.808		0.736		0.713
2020	Liang et al*.* [148]	2.5D CNN and 2.5D CNN	Private (CT)	96	11	–	Left: 0.911; Right: 0.914	0.883	0.868	–	–	0.871	0.874	-
2020	Lei et al*.* [149]	3D R-CNN and 3D U-Net	Private (CT)	15	8	–	0.850	0.820	0.810	–	–	–	–	–
2022	Huang et al*.* [150]	3D CNN and 3D CNN	HNC (CT) [36]	48	9	0.879	0.916	0.884	0.878	0.801	0.776	0.677	0.706	0.643
2022	Huang et al*.* [150]	3D CNN and 3D CNN	StructSeg (CT)	15	7	0.769	0.807	0.802	0.802	–	–	0.499	0.534	0.211
2022	Huang et al*.* [150]	3D CNN and 3D CNN	Private (CT)	15	9	0.957	0.848	0.962	0.946	0.846	0.808	0.824	0.843	0.434
2021	Korte et al*.* [151]	3D U-Net and 3D U-Net	Public RT-MAC dataset (MRI)	31	8	–	–	0.860	0.857	0.830	0.785	–	–	–
2021	Korte et al*.* [151]	3D U-Net and 3D U-Net	Private (MRI)	10	8	–	–	0.730	0.775	0.537	0.435	–	–	–
2021	Gao et al*.* [105]	3D CNN and 3D CC	Private (CT)	1164	22	0.891	Left: 0.924; Right: 0.925	0.846	0.870	–	–	0.713	0.753	0.612
2021	Gao et al*.* [105]	3D CNN and 3D CNN	HNC (CT) [36]	48	9	0.882	0.947	0.898	0.881	0.840	0.838	0.790	0.817	0.713

**Table 3:** DSC-based summary of the paper about abdomen

Year	Refs.	Backbone	Datasets	Scans	Targets	Liver	Spleen	Kidney		Pancreas	Gallbladder	Stomach
								Left	Right			
2017	Gibson et al*.*[66]	3D CNN	TCIA&BTCV [34] (CT)	72	4	0.920	–	–	–	0.660	–	0.830
2017	Men et al*.* [89]	2D CNN	Private (CT)	278	5	–	–	–	–	–	–	–
2018	Shen et al*.* [205]	3D U-Net	Private (CT)	377	7	0.965	0.947	–	–	0.847	0.808	0.963
2018	Gibson et al.[91]	3D V-Net	TCIA&BTCV [34] (CT)	90	8	0.960	0.960	0.950	–	0.780	0.840	0.900
2018	Roth et al*.* [79]	3D U-Net	Private (CT)	377	7	0.971	0.977	–	–	0.849	0.851	0.961
2019	Cai et al*.* [101]	2D FCN	Private (CT)	120	16	0.960	0.951	0.956	0.954	0.785	0.797	0.909
2019	Cai et al*.* [101]	3D GAN	Private (CT)	131 (liver) + 281 (spleen) + 41 (pancreas)	3	0.944	0.960	–	–	0.743	–	–
2019	Heinrich et al*.*[159]	3D U-Net	TCIA(CT)&Private (CT)	43	8	0.954	0.944	–	–	0.702	0.753	0.868
				10	7							
2020	Ahn et al*.* [167]	2.5D CNN	Private (CT)	813 + 150	2	0.973	0.974	–	–	–	–	–
			Private (CT)	813 + 50	2	0.983	0.968	–	–	–	–	–
2020	Fu et al. [93]	3D V-Net	Synapse (CT)	90	8	Aorta, gallbladder, left kidney, right kidney, liver, pancreas, spleen and stomach average DSC: 0.698						
2022	Hatamizadeh et al*.* [127]	3D Transformer and U-Net	BTCV (CT) [32]	30	13	0.983	0.972	0.954	0.942	0.799	0.825	0.945
2020	Chen et al*.* [201]	2.5D U-Net	Private (MR)	102	10	0.963	0.946	0.954	0.954	0.880	0.732	0.923
2020	Tang et al*.* [172]	2.5D U-Net	ABD-110 (CT) [90]	110	11	0.964	0.959	0.96	0.957	0.821	0.822	0.875
2021	Jia and Wei [81]	3D U-Net	CHAOS (CT) [38]	20	4	0.934	0.896	0.937	0.949	–	–	–
2021	Lin et al*.* [190]	3D U-Net	TCIA&BTCV [34] (CT)	90	8	0.953	0.920	0.902	–	0.742	0.760	0.862
2021	Cao et al*.* [114]	2D Transformer	Synapse (CT)	30	8	0.943	0.907	0.833	0.796	0.566	0.665	0.766
2021	Chen et al*.* [126]	2D Transformer And U-Net	Synapse (CT)	30	8	0.941	0.851	0.819	0.770	0.559	0.631	0.756
2022	Song et al*.* [210]	2D CNN	Synapse (CT)	30	8	0.959	0.926	0.906	0.892	0.687	0.671	0.839
2021	Kumar et al*.* [107]	2D GAN	CHAOS (CT) [38]	40	4	Liver, left kidney, right kidney and spleen average DSC: 0.970						
2021	Huang et al*.* [115]	2D Transformer	Synapse (CT)	30	8	0.944	0.919	0.852	0.820	0.657	0.687	0.808
2022	Suo et al*.* [124]	2D Transformer And U-Net	Synapse (CT)	30	8	0.951	0.914	0.890	0.851	0.699	0.720	0.826
2022	Xu et al*.* [92]	3D V-Net	AbdomenCT-1 K [39] + Private (CT)	1112 + 100	4	0.953	0.920	0.914		0.747	–	–
			AbdomenCT-1 K [39] + TCIA& BTCV (CT) [34]	1112 + 90	4	0.961	0.954	0.918	–	0.784	–	–
2022	Berzoini et al*.*[81]	2D U-Net	Open-source CT-org dataset [79]	140	5	0.922	–	0.837		–	–	–
2023	Shen et al*.* [195]	2D U-Net	TCIA (CT)	42	5	0.960	–	–	–	0.754	0.805	0.889
2022	Hong et al*.* [89]	2D U-Net	BTCV [34] &CHAOS [38] (CT)	30 + 20	4	0.884	0.911	0.864	0.891	–	–	–
2021	Xie et al*.* [119]	3D CNN and Transformer	BTCV [34] (CT)	30	11	0.971	0.963	0.939		0.831	0.666	0.882
2022	Srivastava et al*.*[186]	3D U-Net	Synapse (CT)	30	8	0.950	0.870	0.842	0.824	0.681	0.675	0.760
2021	Cross et al*.* [83]	2D U-Net	BTCV (CT) [34]	30	12	0.969	0.958	0.943	0.921	0.798	0.786	0.906
2017	Hu et al*.* [132]	3D FCN and Refinement Model	Private (CT)	140	4	0.960	0.942	0.954	–	–	–	–
2017	Roth et al*.* [158]	3D FCN and 3D FCN	Private (CT)	331	3	0.932	0.906	–	–	0.631	0.706	0.843
2019	Wang et al*.* [171]	2.5D FCN and 2.5D FCN	Private (CT)	236	13	0.980	0.971	0.968	0.984	0.878	0.905	0.952
2020	Zhang et al*.* [133]	3D V-Net and 3D V-Net	BTCV (CT) [34]	30	13	0.945	0.915	0.909	0.919	0.694	0.682	0.784
2020	Xie et al*.* [134]	2.5D FCN and 2.5D FCN	Private (CT)	200	16	0.969	0.968	0.962	0.960	0.877	0.894	0.951
2021	Zhang et al*.* [197]	3D U-Net and 3D U-Net	FLARE 2021 (CT)	511	4	0.954	0.942	0.936	–	0.753	–	–
2021	Lee et al*.* [135]	3D U-Net and 3D U-Net	Private (CT)	100	13	0.960	0.965	0.945	0.920	0.766	0.793	0.833
2018	Kakeya et al*.* [139]	3D U-Net and 3D U-Net	Private (CT)	47	8	0.971	0.969	0.984	0.975	0.861	0.918	-
2018	Larsson et al*.* [152]	Multi-Atlas and 3D FCN	BTCV (CT) [34]	30	13	0.949	0.936	0.911	0.897	0.646	0.613	0.764
2019	Zhao et al*.* [153]	Registration and 2D U-Net	VISCERAL challenge dataset Nonenhanced CT (CTwb)	20	4	–	–	–	–	0.583	0.473	–
2019	Zhao et al. [153]	Registration and 2D U-Net	VISCERAL challenge dataset enhanced CT (CTce)	20	4	–	–	–	–	0.588	0.624	–

**Table 4:** DSC-based summary of the paper about chest

Year	Refs.	Backbone	Datasets	Scans	Targets	Heart	Esophagus	Trachea	Lung		Aorta	Spinal cord
									Left	Right		
2017	Trullo et al*.* [72]	2D FCN	Private (CT)	30	4	0.900	0.670	0.820	–	–	0.860	–
2019	Dong et al*.* [102]	3D GAN	AAPM (CT) [37]	35	4	0.870	0.750	–	0.970	0.970	–	0.900
2020	Vu et al*.* [86]	2D U-Net	Private (CT)	22,411(2D)	5	0.910	0.630	–	0.960	0.960	–	0.710
2019	Lambert et al*.* [45]	2D U-Net	SegTHOR (CT) [38]	60	4	0.930	0.820	0.850	–	–	0.910	–
2019	Vesal et al*.* [182]	2D U-Net	SegTHOR (CT) [38]	60	4	0.941	0.858	0.926	–	–	0.938	–
2020	Shi et al*.* [168]	2.5D U-Net	StructSeg (CT)	50	5	0.941	0.821	0.882	0.968	0.971	–	0.902
2021	Mahmood et al*.* [87]	2D U-Net	AAPM (CT) [37]	60	5	0.880	0.660	–	0.970	0.970	–	0.800
2017	Trullo et al*.* [140]	2D FCN and 2D FCN	Private (CT)	30	4	0.900	0.690	0.870	–	–	0.890	–
2021	Cao et al*.* [141]	2D U-Net and 2D U-Net	SegTHOR (CT)	50	6	0.945	0.850	0.807	0.970	0.966	–	0.910
2020	Zhang et al*.* [133]	3D V-Net and 3D V-Net	SegTHOR (CT)	50	4	0.930	0.785	0.890	–	–	0.916	–
2021	Cao et al*.* [141]	3D U-Net and Two 3D U-Net	AAPM (CT) [37]	60	5	0.941	0.738	–	0.979	0.973	–	0.899
2022	Francis et al*.* [155]	3D U-Net and 3D U-Net	AAPM (CT) [37]	60	5	0.925	0.726	–	0.979	0.972	–	0.893
2022	Francis et al*.* [155]	3D U-Net and 3D U-Net	Private (CT)	30	5	0.860	0.685	–	0.976	0.977	–	0.852

**Table 5:** DSC-based summary of the paper about head and neck-supplementary material

Year	Refs.	Backbone	Datasets	Scans	Targets	Other organs
2017	Ibragimov and Xing [67]	2.5D CNN	Private (CT)	50	13	Pharynx: 0.856; Left eyeball: 0.884; Right eyeball: 0.877; Spinal cord: 0.870; Larynx: 0.693
2019	Van Rooij et al*.* [76]	3D U-Net	Private (CT)	157	11	Larynx: 0.780; Pharyngeal Constrictor: 0.680; Cricopharynx: 0.730; Upper esophageal sphincter: 0.810; Esophagus: 0.600; Oral Cavity: 0.780
2019	Tong et al*.*[100]	3D GAN	Private (MRI)	25	9	Pharynx: 0.706; Larynx: 0.799
2020	Liu et al*.* [82]	3D U-Net	Private (MRI & CT)	45	19	Pharynx: 0.740; Spinal cord: 0.840; Left cochlea: 0.760; Right cochlea: 0.750; Esophagus: 0.850; Oral cavity: 0.900; Left eye: 0.890; Right eye: 0.870; Left lens: 0.730; Right lens: 0.730; Larynx: 0.900; Brain: 0.950
2021	Chen et al.[164]	2.5D U-Net	Private (CT)	307	24	Pituitary: 0.756; Left middle ear: 0.869; Right middle ear: 0.859; Left lens: 0.844; Right lens: 0.839; Left temporomandibular joint: 0.838; Right temporomandibular joint: 0.829
2020	Liu et al*.* [189]	2D U-Net	StructSeg (CT)	50	22	Left eye: 0.858; Right eye: 0.882; Spinal cord: 0.804; Pituitary: 0.503; Left middle ear: 0.825; Right middle ear: 0.717; Left lens: 0.898; Right lens: 0.786; Left temporomandibular joint: 0.723; Right temporomandibular joint: 0.824
2021	Cros et al.[83]	3D U-Net	Private (CT)	200	12	Medullary canal: 0.870; Outer medullary canal: 0.860; Oral cavity: 0.660; Esophagus: 0.600; Trachea: 0.670; Trunk: 0.670; Outer trunk: 0.700; Inner ears: 0.710; Eyes: 0.770; Sub-maxillary glands: 0.740
2021	Lei et al*.* [211]	3D U-Net	StructSeg (CT)	50	22	Left eye: 0.886; Right eye: 0.873; Spinal cord: 0.830; Pituitary: 0.661; Left middle ear: 0.826; Right middle ear: 0.783; Left lens: 0.815; Right lens: 0.754; Left temporomandibular joint: 0.757; Right temporomandibular joint: 0.772
2022	Srivastava et al*.* [186]	3D U-Net	OpenKBP (CT) [40]	188	5	Spinal cord: 0.740
2022	Podobnik et al*.* [95]	2D nnU-net	Private (CT&MRI)	56	31	Spinal cord: 0.812; Pharyngeal constrictor muscles: 0.617; Oral cavity: 0.845; Larynx-supraglottis: 0.728; Larynx-glottis: 0.615; Lips: 0.728; Thyroid: 0.721; Pituitary gland: 0.658; Lacrimal glands (left): 0.621; Lacrimal glands (right): 0.636; Left eye: 0.887; Right eye: 0.884; Left lens: 0.723; Right lens: 0.763; Cervical esophagus: 0.559; Cricopharyngeal inlet: 0.517; Cochleae (left): 0.558; Cochleae (right): 0.514; Carotid arteries (left): 0.624; Carotid arteries (right): 0.618; Buccal mucosa: 0.661; Arytenoids: 0.474
2022	Kan et al*.* [125]	3D Transformer and U-Net	Private (CT)	94	18	Spinal cord: 0.897; Pituitary gland: 0.608; Oral cavity: 0.908; Left eye: 0.907; Right eye: 0.902; Left lens: 0.724; Right lens: 0.689; Left TMJ: 0.789; Right TMJ: 0.778; Left temporal lobe: 0.803; Right temporal lobe: 0.802
2022	Isler et al*.* [181]	2D U-Net	OpenKBP (CT) [40]	188	5	Spinal cord: 0.750
2022	Fang et al*.*[106]	2D FCN and 3D U-Net	Private (CT)	56	14	Right eyeball: 0.634; Left eyeball: 0.636; Lips: 0.676; Oral cavity: 0.829; Throat: 0.389; Esophagus: 0.735; Thyroid gland: 0.642; Spinal cord: 0.782
2021	Gao et al*.*[105]	3D CNN and 3D CNN	Private (CT)	1164	22	Left eye: 0.897; Right eye: 0.895; Left lens: 0.819; Right lens: 0.825; Pituitary gland: 0.722; Left temporal lobe: 0.877; Right temporal lobe: 0.883; Spinal cord: 0.831; Left inner ear: 0.864; Right inner ear: 0.855; Left middle ear: 0.857; Right middle ear: 0.843; Left temporomandibular joint: 0.764; Right temporomandibular joint: 0.789
2018	Larsson et al*.*[152]	Multi-Atlas and 3D FCN	BTCV (CT) [34]	30	13	Esophagus: 0.588; Aorta: 0.870; Inferior vena cava: 0.758; Portal vein and splenic vein: 0.715; Right adrenal gland: 0.630; Left adrenal gland: 0.631
2019	Zhao et al.[153]	Registration and 2D U-Net	VISCERAL challenge dataset enhanced CT (CTce) [185]	20	4	Left adrenal gland: 0.472; Right adrenal gland: 0.390
			VISCERAL challenge dataset enhanced CT (CTce) [185]	20	4	Left adrenal gland: 0.403; Right adrenal gland: 0.434
2018	Men et al*.*[143]	3D U-Net and 3D U-Net	TCIA (CT)	100	7	Spinal cord: 0.910; Left eye: 0.930; Right eye: 0.920
2019	Tang et al*.*[144]	3D U-Net and 3D U-Net	Private (CT)	215	28	Brachial plexus: 0.562; Pharyngeal constrictor: 0.755; Left ear: 0.773; Right ear: 0.786; Left eye: 0.925; Right eye: 0.925; Pituitary gland: 0.639; Larynx: 0.893; Left lens: 0.819; Right lens: 0.830; Oral cavity: 0.908; Spinal cord: 0.856; Sublingual gland: 0.460; Left temporal lobe: 0.848; Right temporal lobe: 0.841; Thyroid: 0.856; Left temporomandibular joint: 0.880; Right temporomandibular joint: 0.869; Trachea: 0.813
2019	Yang et al*.*[145]	3D CNN and 2D U-Net	Private (CT)	88	17	Left eye: 0.875; Right eye: 0.889; Left lens: 0.747; Right lens: 0.698; Cerebellum: 0.936; Pituitary: 0.672; Thyroid: 0.844; Temporal lobe left: 0.762; Temporal lobe right: 0.784; Brain: 0.976; Head: 0.943
2019	Liang et al*.*[146]	2D CNN and 2D CNN	Private (CT)	185	18	Left eye: 0.932; Right eye: 0.936; Left lens: 0.930; Right lens: 0.842; Larynx: 0.870; Oral cavity: 0.928; Left mastoid: 0.821; Right mastoid: 0.824; Spinal cord: 0.884; Left TMJ: 0.846; Right TMJ: 0.844
2019	Gao et al.[147]	3D CNN and 3D CNN	Private (CT)	50	18	Left eye: 0.876; Right eye: 0.912; Oral cavity: 0.792; Larynx: 0.658; Spinal cord: 0.874; Left lens: 0.808; Right lens: 0.790; Pituitary gland: 0.769; Left middle ear: 0.567; Right middle ear: 0.522; Left TMJ:0.584; Right TMJ: 0.572
			Private (CT)	96	11	Left eye: 0.930; Right eye: 0.930; Spinal cord: 0.900; Left lens: 0.872; Right lens: 0.883

**Tabel 6:** DSC-based summary of the paper about abdomen-supplementary material

Year	Refs.	Backbone	Datasets	Scans	Targets	Other organs
2022	Isler et al*.* [181]	2D U-Net	OpenKBP (CT) [40]	188	5	Spinal cord: 0.750
2017	Gibson et al*.* [66]	3D CNN	TCIA &BTCV [34] (CT)	72	4	Esophagus: 0.730
2017	Men et al*.* [89]	2D CNN	Private (CT)	278	5	Bladder: 0.934; Intestine: 0.653; Left femoral head: 0.921; Right femoral head: 0.923; Colon: 0.618
2018	Shen et al*.* [205]	3D U-Net	Private (CT)	377	7	Artery: 0.892; Vein: 0.793
2018	Gibson et al*.* [91]	3D V-Net	TCIA &BTCV [34] (CT)	90	8	Duodenum: 0.630; Esophagus: 0.760
2018	Roth et al*.* [79]	3D U-Net	Private (CT)	377	7	Artery: 0.835; Vein: 0.805
2019	Cai et al*.* [101]	2D FCN	Private (CT)	120	16	Aorta: 0.810; Adrenal gland: 0.368; Celiac AA: 0.385; Duodenum: 0.649; Colon: 0.776; Inferior vena cava: 0.786; Superior mesenteric artery: 0.496; Small bowel: 0.729; Veins: 0.651
2019	Heinrich et al*.*[194]	3D U-Net	TCIA (CT)	43	8	Left adrenal gland: 0.942; Duodenum: 0.538; Esophagus: 0.633
			Private (CT)	10	7	Spleen, Pancreas, Kidney, Gallbladder, Esophagus, Liver, Stomach and duodenum average DSC: 0.823
2022	Hatamizadeh et al*.* [127]	3D Transformer And U-Net	BTCV (CT) [34]	30	13	Esophagus: 0.864; Aorta: 0.948; Inferior vena cava: 0.890; Vein: 0.858
2020	Chen et al*.* [165]	2.5D U-Net	Private (MR)	102	10	Duodenum: 0.801; Small intestine: 0.870; Spinal cord: 0.904; Vertebral body: 0.900
2020	Tang et al*.* [172]	2.5D U-Net	ABD-110 (CT)	110	11	Large intestine: 0.825; Small intestine: 0.765; Duodenum: 0.707; Spinal cord: 0.908
2021	Jia and Wei [80]	3D U-Net	CHAOS (CT) [38]	20	4	-
2021	Lin et al*.* [190]	3D U-Net	TCIA &BTCV(CT) [29]	90	8	Duodenum: 0.637; Esophagus: 0.733
2020	Fu et al*.* [93]	2D Transformer	Synapse (CT)	30	8	Aorta: 0.855
2021	Chen et al*.* [126]	2D Transformer And U-Net	Synapse (CT)	30	8	Aorta: 0.872
2022	Song et al*.* [210]	2D CNN	Synapse (CT)	30	8	Aorta: 0.903
2021	Huang et al*.* [115]	2D Transformer	Synapse (CT)	30	8	Aorta: 0.870
2022	Suo et al*.* [124]	2D Transformer And U-Net	Synapse (CT)	30	8	Aorta: 0.881
2022	Berzoini et al*.*[81]	2D U-Net	Open-source CT-org datase	140	6	Lung: 0.967; Bladder: 0.836; Bone: 0.944
2023	Shen et al*.* [195]	2D U-Net	TCIA (CT)	42	5	Duodenum: 0.615
2021	Xie et al*.* [119]	3D CNN and Transformer	BTCV (CT) [34]	30	11	Esophagus: 0.780; Aorta: 0.912; Inferior vena cava: 0.880; Portal vein and splenic vein: 0.781
2022	Srivastava et al*.*[186]	3D U-Net	Synapse (CT)	30	8	Aorta: 0.909
2022	Jiang et al*.* [84]	2D U-Net	BTCV (CT) [34]	30	12	Esophagus: 0.807; Aorta: 0.913; Inferior vena cava: 0.850; Portal vein and splenic vein: 0.809; Adrenal gland: 0.691
2017	Roth et al*.*[158]	3D FCN and 3D FCN	Private (CT)	331	3	Artery: 0.796; Vein: 0.731
2019	Wang et al*.*[171]	2.5D FCN and 2.5D FCN	Private (CT)	236	13	Aorta: 0.918; Colon: 0.830; Duodenum: 0.754; Inferior vena cava: 0.870; Small intestine: 0.801; Vein: 0.807
2020	Zhang et al*.* [133]	3D V-Net and 3D V-Net	BTCV (CT) [34]	30	13	Esophagus: 0.691; Aorta: 0.877; Inferior vena cava: 0.865; Portal vein and splenic vein: 0.688; Right adrenal gland: 0.651; Left adrenal gland: 0.619
2020	Xie et al*.*[134]	2.5D FCN and 2.5D FCN	Private (CT)	200	16	Aorta: 0.937; Adrenal gland: 0.630; Abdominal cavity: 0.620; Duodenum: 0.735; Inferior vena cava: 0.837; Vascular: 0.742; Small intestine: 0.751; Vein: 0.748; Colon: 0.800
2021	Lee et al*.*[135]	3D U-Net and 3D U-Net	BTCV (CT) [34]	47	8	Esophagus: 0.783; Aorta: 0.916; Inferior vena cava: 0.856; Portal vein and splenic vein: 0.762; RAD: 0.741; LAD: 0.746
2018	Kakeya et al*.* [139]	3D U-Net and 3D U-Net	Private (CT)	47	8	Inferior vena cava: 0.908; Aorta: 0.969
2018	Men et al*.*[143]	3D U-Net and 3D U-Net	TCIA (CT)	100	7	Spinal cord: 0.910; Left eye: 0.930; Right eye: 0.920
2020	Lei et al*.* [149]	3D R-CNN and 3D U-Net	Private (CT)	15	8	Esophagus: 0.840; Throat: 0.790; Oral: 0.890; Pharynx: 0.850; Spinal cord: 0.890
2021	Korte et al*.*[151]	3D U-Net and 3D U-Net	Public RT-MAC dataset (MRI)	43	8	Secondary lymph nodes (left): 0.708; Secondary lymph nodes (right): 0.715; Tertiary lymph nodes (left): 0.561; Tertiary lymph nodes (right): 0.573
			Private (MRI)	10	8	Secondary lymph nodes (left): 0.553; Secondary lymph nodes (right): 0.525; Tertiary lymph nodes (left): 0.304; Tertiary lymph nodes (right): 0.189

### Single network

#### CNN-based methods

CNN can automatically extract features from input image. Multiple neurons are connected to each neuron in next layer, where each layer can perform tasks such as convolution, pooling or loss computation [63]. CNNs have been successfully applied to medical images, such as brain [64, 65] and pancreas [66] segmentation tasks.

#### Early CNN-based methods

Earlier CNN-based methods mainly utilized convolutional layers for feature extraction, followed by pooling layers and fully connected layers for final prediction. In the work of Ibragimov and Xing [67], deep learning techniques were employed for the segmentation of OARs in head and neck CT images for the first time. They trained 13 CNNs for 13 OARs and demonstrated that the CNNs outperformed or were comparable to advanced algorithms in accurately segmenting organs such as the spinal cord, mandible and optic nerve. However, they did not perform well in segmenting organs such as the optical chiasm. Fritscher et al*.* [68] incorporated shape location and intensity information with CNN for segmenting the optic nerve, parotid gland, and submandibular gland. Moeskops et al*.* [69] investigated whether a single CNN can be used for segmenting multiple tissues across different modalities, including six tissues in brain MR images, pectoral muscles in breast MR images, and coronary arteries in heart CTA images. Their results demonstrated that a single CNN can effectively segment multiple organs across different imaging modalities.

#### FCN-based methods

Early methods based on CNN showed some improvement in segmentation accuracy compared to traditional methods. However, CNN involves multiple identical computations of overlapping voxels during the convolution operation, which may cause some performance loss. Moreover, the final fully connected network layer in CNN can introduce spatial information loss to the image. To overcome these limitations, Shelhamer et al*.* [70] proposed the Fully Convolutional Network (FCN), which utilized transposed convolutional layers to achieve end-to-end segmentation while preserving spatial information. Wang et al*.* [71] used FCN with a novel sample selection strategy to segment 16 organs in the abdomen, while Trullo et al*.* [72] employed a variant of FCN called SharpMask [73] to enhance the segmentation performance of 5 organs in the thorax compared to standard FCN.

#### U-Net-based methods

The U-Net architecture, proposed by Ronneberger et al*.* [74], builds upon the FCN framework and consists of an encoder and a decoder, connecting them layer by layer with skip connections that allow for multiscale feature fusion. U-Net has become a widely adopted architecture in multi-organ segmentation [75–89]. For example, Roth et al*.* [79] employed U-Net to segment 7 organs in the abdomen with an average Dice value of 0.893. Lambert et al*.* [45] proposed a simplified U-Net for segmenting the heart, trachea, aorta, and esophagus of the chest, which improved performance by adding dropout and bilinear interpolation. Apart from U-Net, V-Net [90] introduced a volumetric, fully convolutional neural network for 3D image segmentation [91–93]. Gibson et al*.* [91] used dense V-Networks to segment 8 organs in the abdomen, while Xu et al*.* [92] proposed a probabilistic V-Net model with a conditional variational autoencoder (cVAE) and hierarchical spatial feature transform (HSPT) for abdominal organs segmentation. The nnU-Net [94] is a novel framework based on U-Net architecture with adaptive pre-processing, data enhancement, and postprocessing techniques, which has demonstrated state-of-the-art performance in various biomedical segmentation challenges [95–98]. Podobnik et al*.* [95] reported successful results in segmenting 31 OARs in the head and neck using nnU-Net, with both CT and MR images being employed.

#### GAN-based methods

GAN [99] usually comprises a pair of competitive networks: generators and discriminators. The generator attempts generate synthetic data that can deceive the discriminator, while the discriminator strives to accurately distinguish between real and generated data. After iterative optimization training, the performance of both networks can be improved. In recent years, several GAN-based multi-organ segmentation methods have been proposed and achieved high segmentation accuracy [100–107].

Dong et al*.* [102] employed a GAN framework with a set of U-Nets as the generator and a set of FCNs as the discriminator to segment the left lung, right lung, spinal cord, esophagus and heart from chest CT images. The results showed that the adversarial networks enhanced the segmentation performance of most organs, with average DSC values of 0.970, 0.970, 0.900, 0.750, and 0.870 for the above five organs. Tong et al*.* [100] proposed a Shape-Constraint GAN (SC-GAN) for automatic segmentation of head and neck OARs from CT and low-field MR images. It used DenseNet [108], a deep supervised fully convolutional network, to segment organs for prediction and uses a CNN as the discriminator network to correct the prediction errors. The results showed that combining GAN and DenseNet could further improve the segmentation performance of CNN by incorporating original shape constraints.

While GAN can enhance accuracy with its adversarial losses, training a GAN network is challenging and time-consuming since the generator must achieve Nash equilibrium with the discriminator [99]. Moreover, its adversarial loss, as a shape modifier, can only achieve higher segmentation accuracy when segmenting organs with regular and distinctive shapes (e.g., liver and heart) but may not work well for irregular or tubular structures (such as the pancreas and aorta) [109].

#### Transformer-based methods

CNN-based methods have demonstrated impressive effectiveness in segmenting multiple organs across various tasks. However, a significant limitation arises from the inherent shortcomings of the limited perceptual field within the convolutional layers. Specifically, these limitations prevent CNNs from effectively modeling global relationships. This constraint impairs the models' overall performance by limiting their ability to capture and integrate broader contextual information which is critical for accurate segmentation. The self-attention mechanism of transformer [32] can overcome the long-term dependency problem and achieve superior results compared to CNNs in several tasks, including natural language processing and computer vision. In recent studies, it has been demonstrated that medical image segmentation networks employing transformers can achieve comparable or superior accuracy compared to current state-of-the-art methods [110–113].

For instance, Cao et al*.* [114] incorporated the transformer into a U-shaped network, named Swin-UNet, to investigate the effectiveness of the pure transformer model in abdominal multi-organ segmentation, which showed promising segmentation accuracy. However, this method requires initializing the network encoder and decoder with the training weights of the Swin transformer on ImageNet. Huang et al*.* [115] introduced MISSFormer, a novel architecture for medical image segmentation that addresses convolution's limitations by incorporating an Enhanced Transformer Block. This innovation enables effective capture of long-range dependencies and local context, significantly improving segmentation performance. Furthermore, in contrast to Swin-UNet, this method can achieve comparable segmentation performance without the necessity of pre-training on extensive datasets. Tang et al*.*[116] introduce a novel framework for self-supervised pre-training of 3D medical images. This pioneering work includes the first-ever proposal of transformer-based pre-training for 3D medical images, enabling the utilization of the Swin Transformer encoder to enhance fine-tuning for segmentation tasks.

While transformer-based methods can capture long-range dependencies and outperform CNNs in several tasks, they may struggle with the detailed localization of low-resolution features, resulting in coarse segmentation results. This concern is particularly significant in the context of multi-organ segmentation, especially when it involves the segmentation of small-sized organs [117, 118].

#### Hybrid networks

CNNs are proficient at detecting local features but frequently struggle to capture global features effectively. In contrast, transformers can capture long-range feature dependencies but may lose local feature details and result in poor segmentation accuracy for small organs. To overcome the limitations, researchers have explored hybrid methods that combine CNN and transformer frameworks [111, 119–123].

For example, Suo et al*.* [124] proposed the I2-Net, a collaborative learning network that combines features extracted by CNNs and transformers to accurately segment multiple abdominal organs. This method resulted in an enhancement of the segmentation accuracy for small organs by 4.19%, and for medium-sized organs by a range of 1.83% to 3.8%. Kan et al*.* [125] proposed ITUnet, which added transformer-extracted features to the output of each block of the CNN-based encoder, obtaining segmentation results that leveraged both local and global information. ITUnet demonstrated better accuracy and robustness than other methods, especially on difficult organs such as the lens. Chen et al*.* [126] introduced TransUNet, a network architecture that utilized transformers to build stronger encoders and competitive results for head and neck multi-organ segmentation. Similarly, Hatamizadeh et al. [127] introduced UNETR and Swin UNETR [128], which employed transformers (Swin transformer) as encoders and CNNs as decoders. This hybrid method captured both global and local dependencies, leading to improved segmentation accuracy.

In addition to the methods combining CNN and transformer, there are some other hybrid architectures. For example, Chen et al*.* [129] integrated U-Net with long short-term memory (LSTM) for chest organ segmentation, and the DSC values of all five organs were above 0.8. Chakravarty et al*.* [130] introduced a hybrid architecture that leveraged the strengths of both CNNs and recurrent neural networks (RNNs) to segment the optic disc, nucleus, and left atrium. The hybrid methods effectively merge and harness the advantages of both architectures for accurate segmentation of small and medium-sized organs, which is a crucial research direction for the future.

#### Cascade network

Segmenting small organs in medical images is challenging because most organs occupy only a small volume in the images, making it difficult for segmentation models to accurately identify them. To address this constraint, researchers have proposed cascade multi-stage methods, which can be categorized into two types. One is coarse-to-fine-based method [131–141], where the first network is utilized to acquire a coarse segmentation, followed by the second network that refines the coarse outcomes for improved accuracy. The other is localization and segmentation-based method [105, 142–153], where registration methods or localization networks are used to identify candidate boxes for the location of each organ, which are then input into the segmentation network, which is shown in Fig. 7 (B). Additionally, the first network can provide other information, including organ shape, spatial location, or relative proportions, to enhance the segmentation accuracy of the second network.

#### Coarse-to-fine-based methods

The coarse-to-fine-based methods first input the original image and its corresponding labels into the first network to obtain probability map. This probability map will multiply the original image and be input into the second network to refine the coarse segmentation, as illustrated in Fig. 7(A). Over the years, numerous methods utilizing the coarse-to-fine method have been developed for multi-organ segmentation, with references in [131–141].

Trullo et al*.* [72] proposed 2 deep architectures that work synergistically to segment several organs such as the esophagus, heart, aorta, and trachea. In the first stage, probabilistic maps were obtained to learn anatomical constrains. Then, four networks were trained to distinguish each target organ from the background in separate refinements. Zhang et al*.* [133] developed a new cascaded network model with Block Level Skip Connections (BLSC) between two networks, allowing the second network to benefit from the features learned by each block in the first network. By leveraging these skip connections, the second network can converge more quickly and effectively. Xie et al*.* [134] proposed a new framework named the Recurrent Saliency Transformation Network (RSTN) which used coarse segmentation masks as spatial weights in the fine stage, effectively guiding the network's attention to important regions for accurate segmentation. Moreover, by enabling gradients to be backpropagated from the loss layer to the entire network, the RSTN facilitates joint optimization of the two stages. Ma et al*.* [154] presented a comprehensive coarse-to-fine segmentation model for automatic segmentation of multiple OARs in head and neck CT images. This model used a predetermined threshold to classify the initial results of the coarse stage into large and small OARs, and then designed different modules to refine the segmentation results.

This coarse-to-fine method efficiently simplifies the background and enhances the distinctiveness of the target structures. By dividing the segmentation task into two stages, this method achieves better segmentation results for small organs compared to the single-stage method. Nevertheless, it is essential to acknowledge that this method entails certain limitations, including heightened memory usage and extended training times attributed to the necessity of train at least two networks.

#### Localization and segmentation-based methods

In the localization and segmentation-based method, the first network provides location information and generates a candidate frame, which is then used to extract the Region of Interests (ROIs) from the image. This extracted region, free from interference of other organs or background noise, serves as the input for the second network. By isolating the targeted organ, the segmentation accuracy is improved. The process is illustrated in Fig. 7(B). The organ location in the first stage can be obtained through registration or localization network, with reference in [105, 142–153].

Wang et al*.* [142], Men et al*.* [143], Lei et al*.* [149], Francis et al*.* [155], and Tang et al*.* [144] used neural networks in both stages. In the first stage, networks were used to localize the target OARs by generating bounding boxes. In the second stage, the target OARs were segmented within the bounding boxes. Among them, Wang et al*.* [142] and Francis et al*.* [155] utilized 3D U-Net in both stages, while Lei et al*.* [149] used Faster RCNN to automatically locate the ROI of organs in the first stage. Furthermore, FocusNet [105, 147] presented a novel neural network that effectively addresses the challenge of class imbalance in the segmentation of head and neck OARs. The small organs are first localized using the organ localization network, and then high-resolution features of small organs are fed into the segmentation network. Liang et al*.* [146] introduced a multi-organ segmentation framework that utilizes multi-view spatial aggregation to integrate the learning of both organ localization and segmentation subnetworks. This framework mitigates the impact of neighboring structures and background regions in the input data, and the proposed fine-grained representation based on ROIs enhances the segmentation accuracy of organs with varying sizes, particularly small organs.

Larsson et al*.* [152], Zhao et al*.* [153], Ren et al*.* [156], and Huang et al*.* [150] utilized registration-based methods to localize organs, while CNN was employed for accurate segmentation. Ren et al*.* [156] used interleaved cascades of 3D-CNNs to segment each organ, exploiting the high correlation between adjacent tissues. Specifically, the initial segmentation results of a particular tissue can improve the segmentation of its neighboring tissues. Zhao et al*.* [153] proposed a flexible knowledge-assisted framework that synergistically integrated deep learning and traditional techniques to improve segmentation accuracy in the second stage.

Localization and segmentation-based methods have proven to enhance the accuracy of organ segmentation by reducing background interference, particularly for small organs. However, this method requires considerable memory and training time, and the accuracy of segmentation is heavily reliant on the accuracy of organ localization. Therefore, improving the localization of organs and enhancing segmentation accuracy are still areas of research that need further exploration in the future.

#### Other cascade methods

In addition to probability maps and localization information, the first network can also provide other types of information that can be used to improve segmentation accuracy, such as scale information and shape priors. For instance, Tong et al*.* [157] combined FCNN with a shape representation model (SRM) for head and neck OARs segmentation. The SRM serves as the first network for learning highly representative shape features in head and neck organs, which are then used to improve the accuracy of the FCNN. The results from comparing the FCNN with and without SRM indicated that the inclusion of SRM greatly raised the segmentation accuracy of 9 organs, which varied in size, morphological complexity, and CT contrasts. Roth et al*.* [158] proposed two cascaded FCNs, where low-resolution 3D FCN predictions were upsampled, cropped, and connected to higher-resolution 3D FCN inputs.

#### Step-by-step segmentation network

In the context of multi-organ segmentation, step-by-step segmentation refers to sequentially segmenting organs in order of increasing complexity, starting with easier-to-segment organs before moving on to more challenging ones, which is shown in Fig. 7(C). The fundamental assumption is that segmenting more challenging organs (e.g., those with more complex shapes and greater variability) can benefit from the segmentation results of simpler organs processed earlier [159]. Step-by-step segmentation has been demonstrated to be highly effective for segmenting some of the most challenging organs, such as the pancreas (Hammon et al. [160]), utilizing surrounding organs (such as the liver and spleen) as supportive structures.

In recent years, many deep learning-based step-by-step segmentation methods have emerged. For example, Zhao et al*.* [161] first employed the nnU-Net to segment the kidneys and then to segment kidney tumors based on the segmentation results of the kidneys. Similarly, Christ et al*.*[136] first segment the liver, followed by the segmentation of liver tumors based on the segmentation results of the liver. In [162], organs susceptible to segmentation errors, such as the lungs, are segmented first, followed by the segmentation of less susceptible organs, such as airways, based on lung segmentation. Guo et al*.* [163] proposed a method called Stratified Organ at Risk Segmentation (SOARS), which categorizes organs into anchor, intermediate, and small and hard (S&H) categories. Each OAR category uses a different processing framework. Inspired by clinical practice, anchor organs are utilized to guide the segmentation of intermediate and S&H category organs.

### Network dimension

Considering the dimension of input images and convolutional kernels, multi-organ segmentation networks can be divided into 2D, 2.5D and 3D architectures, and the differences among three architectures will be discussed in follows.

### 2D- and 3D-based methods

The 2D multi-organ segmentation network takes as input slices from a three-dimensional medical image, and the convolution kernel is also two-dimensional. Several studies, including those by Men et al*.* [89], Trullo et al*.* [72], Gibson et al*.* [91], Chen et al*.* [164], Zhang et al*.* [78], and Chen et al*.* [165], have utilized 2D networks for multi-organ segmentation. 2D architectures can reduce the GPU memory burden. But CT or MR images are inherently 3D, slicing images into 2D tends to ignore the rich information in the entire image voxel, so 2D models are insufficient for analyzing the complex 3D structures in medical images.

3D multi-organ segmentation networks can extract features directly from 3D medical images by using 3D convolutional kernels. Some studies, such as Roth et al*.*[79], Zhu et al*.* [75], Gou et al*.* [77], and Jain et al*.* [166], have employed 3D network for multi-organ segmentation. However, since 3D network requires a large amount of GPU memory, they may face computationally intensive and memory shortage problems. As a result, most 3D network-based methods use sliding windows acting on patches. To overcome the constraints of GPU memory, Zhu et al*.* [75] proposed a model called AnatomyNet, which took full-volume of head and neck CT images as inputs and generated masks for all organs to be segmented at once. To balance GPU memory usage and network learning capability, they employed a down-sampling layer solely in the first encoding block, which also preserved information of small anatomical structures.

### Multi-view-based methods

Accurate medical image segmentation requires effective use of spatial information among image slices. Inputting 3D images directly to the neural network can lead to high memory usage, while converting 3D images to 2D slices results in the loss of spatial information between slices. As a solution, multi-view-based methods have been proposed, which include using 2.5D neural networks with multiple 2D slices or combining 2D and 3D convolutions. This method can reduce memory usage while maintaining the spatial information between slices, improving the accuracy of medical image segmentation.

**Fig. 8:**
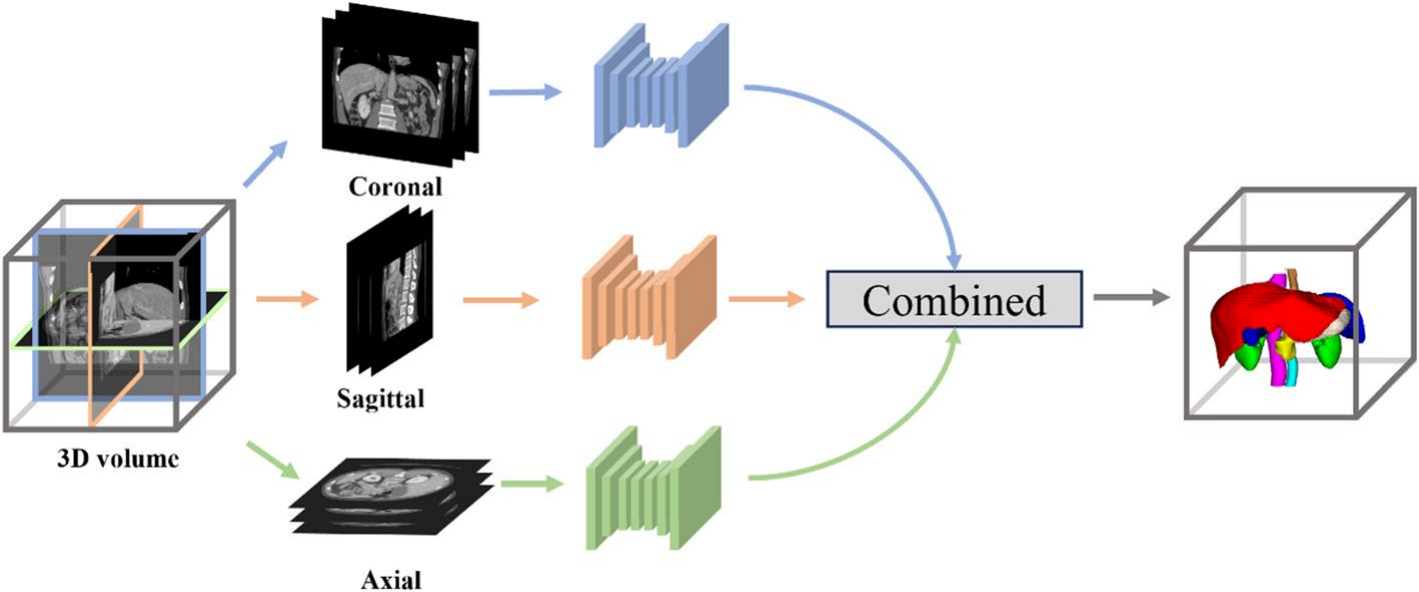
Framework of multi-view-based methods

The 2.5D-based method uses 2D convolutional kernels and takes in multiple slices as input. The slices can either be a stack of adjacent slices using interslice information [167, 168], or slices along three orthogonal directions (axial, coronal, and sagittal) [67, 68, 148, 169], which is shown in Fig. 8. Zhou et al*.* [170] segmented each 2D slice using FCN by sampling a 3D CT case on three orthogonally oriented slices and then assembled the segmented output (i.e., 2D slice results) back into 3D. Chen et al*.* [165] developed a multi-view training method with a majority voting strategy. Wang et al*.* [171] used a statistical fusion method to combine segmentation results from three views. Liang et al*.* [148] performed context-based iterative refinement training on each of the three views and aggregated all the predicted probability maps to obtain final segmentation results. These methods have shown improved segmentation results compared to the three separate views.

Tang et al*.* [172] proposed a novel method which combines the strengths of 2D and 3D models. This method utilized high-resolution 2D convolution for accurate segmentation and low-resolution 3D convolution for extracting spatial contextual information. A self-attention mechanism controlled the corresponding 3D features to guide 2D segmentation, and experiments demonstrated that this method outperforms both 2D and 3D models. Similarly, Chen et al*.* [164] devised a novel convolutional neural network, OrganNet2.5D, that effectively processed diverse planar and depth resolutions by fully utilizing 3D image information. This network combined 2D and 3D convolutions to extract both edge and high-level semantic features.

Some studies only used 2D images to avoid memory and computation problems, but they did not fully exploit the potential of 3D image information. Although 2.5D methods can make better use of multiple views, their ability to extract spatial contextual information is still limited. Current 2.5D methods in multi-organ segmentation aggregate three perspectives at the outcome level, but the intermediate processes are independent of each other, and more effective use of intermediate learning processes is an area for further investigation. Pure 3D networks have a high parameter and computational burden, which limits their depth and performance. As for this reason, some people have begun researching lightweight 3D networks, Zhao et al*.*[173] proposed a novel framework based on lightweight network and Knowledge Distillation (KD) for delineating multiple organs from 3D CT volumes. Thus, finding better ways to combine multi-view information to achieve accurate multi-organ segmentation while considering memory and computational resources is a promising research direction.

### Image segmentation modules

The design of network architecture is a crucial factor in improving the accuracy of multi-organ segmentation, but the process of designing such a network is quite intricate. In multi-organ segmentation tasks, various special mechanisms, such as dilation convolution module, feature pyramid module, and attention module, have been developed to enhance the accuracy of organ segmentation. These modules increase the perceptual field, combine features of different scales, and concentrate the network on the segmented region, thereby enhancing the accuracy of multi-organ segmentation. Cheng et al*.* [174] have explored the efficacy of each module in the network compared with the basic U-Net network for the head and neck segmentation task.

### Shape prior module

Shape prior has been shown to be particularly effective for medical images due to the fixed spatial relationships between internal structures. As a result, incorporating anatomical priors in multi-organ segmentation task can significantly enhance the segmentation performance.

There are two main methods used for incorporating anatomical priors in multi-organ segmentation tasks. The first method is based on statistical analysis, which involves calculating the average distribution of organs in a fully labeled dataset. The segmentation network predictions are then guided to be as close as possible to this average distribution of organs [66, 68, 102, 175, 176]. The second method involves training a shape representation model that is pretrained using annotations from the training dataset. This model is used as a regularization term to constrain the predictions of the network during training [100, 157]. For example, Tappeiner et al*.*[177] propose that using stacked convolutional autoencoders as shape priors can enhance segmentation accuracy, both on small datasets and complete datasets. Recently, it has been demonstrated that generative models such as diffusion models [178, 179] can learn anatomical priors [180]. Therefore, utilizing generative models to obtain anatomical prior knowledge is a promising future research direction for improving segmentation performance.

### Dilated convolutional module

In conventional CNN, down-sampling and pooling operations are commonly employed to expand the perception field and reduce computation, but these can cause spatial information loss and hinder image reconstruction. Dilated convolution (also referred to as "Atrous") introduces an additional parameter, expansion rate, to the convolution layer, which can allow for the expansion of the perception field without increasing computational cost. Dilated convolution is widely used in multi-organ segmentation tasks [66, 80, 168, 181, 182] to enlarge the sampling space and enable the neural network to extract multiscale contextual features across a wider receptive field. For instance, Li et al*.*[183] proposed a high-resolution 3D convolutional network architecture that integrates dilated convolutions and residual connections to incorporates large volumetric context. The effectiveness of this approach has been validated in brain segmentation tasks using MR images. Gibson et al*.* [66] utilized CNN with dilated convolution to accurately segment organs from abdominal CT images. Men et al*.* [89] introduced a novel Deep Dilated Convolutional Neural Network (DDCNN) for rapid and consistent automatic segmentation of clinical target volumes (CTVs) and OARs. Vesal et al*.* [182] integrated dilated convolution into the 2D U-Net for segmenting esophagus, heart, aorta, and thoracic trachea.

### Multiscale module

Neural networks are composed of layers that progressively extract features from input data. The lower layers capture fine-grained geometric details with a smaller receptive field, providing high-resolution but weaker semantic representation. Conversely, higher layers have a larger receptive field and stronger semantic representation, but lower feature map resolution, which may cause information loss for small targets. To address this, multiscale fusion modules have been proposed, including bottom-up, top-down, and lateral feature pyramids (FPNs) [184], spatial pooling pyramids (ASPPs) [185] that combine dilated convolution and multiscale fusion. In multi-organ segmentation tasks, multiscale feature fusion is widely used because of the different sizes of organs. For example, Jia and Wei [80] introduced the feature pyramid into a multi-organ segmentation network using two opposite feature pyramids (top-down and bottom-up) to handle multiscale changes and improve the segmentation accuracy of small targets. Shi et al*.* [168] used the pyramidal structure of lateral connections between encoders and decoders to capture contextual information at multiple scales. Additionally, Srivastava et al*.* [186] introduced OARFocalFuseNet, a novel segmentation architecture that utilized a focal modulation scheme for aggregating multiscale contexts in a dedicated resolution stream during multiscale fusion.

### Attention module

The attention module is a powerful tool that allows the network to dynamically weight important features. It can leverage the inherent self-attentiveness of the network and is especially useful for multi-organ segmentation tasks [101, 187]. There are several kinds of attention mechanisms, such as channel attention, spatial attention, and self-attention, which can be used to selectively emphasize the most informative features.

Squeeze-and-excitation (SE) module [188] is an effective channel attention technique that enables the network to emphasize important regions in an image. AnatomyNet [75] utilized 3D SE residual blocks to segment the OARs in the head and neck. This method enabled the extraction of 3D features directly from CT images and dynamically adjusted the mapping of residual features within each channel by generating a channel attention tensor. Liu et al*.* [189] proposed a novel network architecture, named Cross-layer Spatial Attention map Fusion CNN (CSAF-CNN), which could integrate the weights of different spatial attentional maps in the network, resulting in significant improvements in segmentation performance. In particular, the average DSC of 22 organs in the head and neck was 72.50%, which outperformed U-Net (63.9%) and SE-UNet (67.9%). Gou et al*.* [77] designed a Self-Channel-Spatial-Attention neural network (SCSA-Net) for 3D head and neck OARs segmentation. This network could adaptively enhance both channel and spatial features, and it outperformed SE-Res-Net and SE-Net in segmenting the optic nerve and submandibular gland. Lin et al*.* [190] proposed a variance-aware attention U-Net network that embedded variance uncertainty into the attention architecture to improve the attention to error-prone regions (e.g., boundary regions) in multi-organ segmentation. This method significantly improved the segmentation results of small organs and organs with irregular structures (e.g., duodenum, esophagus, gallbladder, and pancreas). Zhang et al*.* [78] proposed a novel network called Weaving Attention U-Net (WAU-Net) that combined the U-Net +  + [191] with axial attention blocks to efficiently model global relationships at different levels of the network. This method achieved competitive performance in segmenting OARs of the head and neck.

### Other modules

The dense block [108] can efficiently use the information of the intermediate layer, and the residual block [192] can prevent gradient disappearance during backpropagation. These two modules are often embedded in the basic segmentation framework. The convolution kernel of the deformable convolution [193] can adapt itself to the actual situation and better extract features. Heinrich et al*.* [194] proposed the OBELISK-Net, a 3D abdominal multi-organ segmentation architecture that incorporated sparse deformable convolutions with conventional CNNs to enhance segmentation of small organs with large shape variations such as the pancreas and esophagus. The deformable convolutional block proposed by Shen et al*.* [195] can handle shape and size variations across organs by generating specific receptive fields with trainable offsets. The strip pooling [196] module targets long strip structures (e.g., esophagus and spinal cord) by using long pooling instead of square pooling to avoid contamination from unrelated regions and capture remote contextual information. For example, Zhang et al*.* [197] utilized a pool of anisotropic strips with three directional receptive fields to capture spatial relationships between multiple organs in the abdomen. Compared to network architectures, network modules have gained widespread use due to their simple design process and ease of integration into various architectures.

### Loss function

It is widely recognized that the choice of loss function is of vital importance in determining the segmentation accuracy. In multi-organ segmentation tasks, choosing an appropriate loss function can address the class imbalance issue and improve the segmentation accuracy of small organs. Jadon [198] has provided a comprehensive overview of commonly used loss functions in semantic segmentation; Ma et al*.*[199] systematically summarized common loss functions used in medical image segmentation and evaluated the effectiveness of each loss function across multiple datasets. In the context of multi-organ segmentation, commonly used loss functions include CE loss [200], Dice loss [201], Tversky loss [202], focal loss [203], and their combinations.

### CE loss

The CE loss (cross-entropy loss) [200] is a widely used information theoretic measure that compares the predicted output labels with the ground truth. Men et al*.* [89], Moeskops et al*.* [95], and Zhang et al*.* [78] utilized CE loss for multi-organ segmentation. However, in situations where the background pixels greatly outnumber the foreground pixels, CE loss can result in poor segmentation outcomes by heavily biasing the model towards the background. To overcome this issue, the weighted CE loss [204] added weight parameters to each category based on CE loss, making it better suited for situations with unbalanced sample sizes. Since multi-organ segmentation often faces a significant class imbalance problem, using the weighted CE loss is a more effective strategy than using only the CE loss. As an illustration, Trullo et al*.* [72] used a weighted CE loss to segment the heart, esophagus, trachea, and aorta in chest images, while Roth et al*.* [79] applied a weighted CE loss for abdomen multi-organ segmentation.

### Dice loss

Milletari et al*.* [90] proposed the Dice loss to quantify the intersection between volumes, which converted the voxel-based measure to a semantic label overlap measure, becoming a commonly used loss function in segmentation tasks. Ibragimov and Xing [67] used the Dice loss to segment multiple organs of the head and neck. However, using the Dice loss alone does not completely solve the issue that neural networks tend to perform better on large organs. To address this, Sudre et al*.* [201] introduced the weighted Dice score (GDSC), which adapted its Dice values considering the current class size. Shen et al*.* [205] assessed the impact of class label frequency on segmentation accuracy by evaluating three types of GDSC (uniform, simple, and square). Gou et al*.* [77] employed GDSC for head and neck multi-organ segmentation, while Tappeiner et al*.* [206] introduced a class-adaptive Dice loss based on nnU-Net to mitigate high imbalances. The results showcased the method's effectiveness in significantly enhancing segmentation outcomes for class-imbalanced tasks. Kodym et al. [207] introduced a new loss function named as the batch soft Dice loss function for training the network. Compared to other loss functions and state-of-the-art methods on current datasets, models trained with batch Dice loss achieved optimal performance.

### Other losses

The Tversky loss [202] is an extension of the Dice loss and can be fine-tuned by adjusting its parameters to balance the rates of false positives and false negatives. The focal loss [203] was originally proposed for object detection to highlight challenging samples during training. Similarly, the focal Tversky loss [208] assigns less weight to easy-to-segment organs and focuses more on difficult organs. Berzoini et al*.* [81] applied the focal Tversky loss to smaller organs, which balances the performance between organs of different sizes and assigns more weight to hard-to-segment small organs, thus solving the class imbalance issue caused by kidneys and bladders. Inspired by the exponential logarithmic loss (ELD-Loss) [209], Liu et al*.* [189] introduced the top-k exponential logarithmic loss (TELD-Loss) to address the issue of class imbalance in head and neck OARs segmentation. Results indicate that the TELD-Loss is a robust method, particularly when dealing with mislabeling problems.

### Combined loss

To address the advantages and disadvantages of different loss functions in multi-organ segmentation, researchers have proposed combining multiple loss functions for improved outcomes. The commonly employed method is a weighted sum of Dice loss and CE loss. Dice loss tackles class imbalance, while CE loss enhances curve smoothing. For instance, Isensee et al*.* [94] introduced a hybrid loss function that combines Dice loss and CE loss to calculate the similarity between predicted voxels and ground truth. Several other studies, including Isler et al*.* [181], Srivastava et al*.* [186], Xu et al*.* [92], Lin et al*.* [190], and Song et al*.* [210], have also adopted this weighted combination loss for multi-organ segmentation. Zhu et al*.* [75] specifically studied different loss functions for the unbalanced head and neck region and found that combining Dice loss with focal loss was superior to using the ordinary Dice loss alone. Similarly, both Cheng et al*.* [174] and Chen et al*.* [164] have used this combined loss function in their studies.

Conventional Dice loss may not effectively handle smaller structures, as even a minor misclassification can greatly impact the Dice score. Lei et al*.* [211] introduced a novel hardness-aware loss function that prioritizes challenging voxels for improved segmentation accuracy. Song et al*.* [212] proposed a dynamic loss weighting algorithm that dynamically assigns larger loss weights to organs that are classified as more difficult to segment based on data and network state, forcing the network to learn more from these organs, thereby maximizing segmentation performance. Designing an appropriate loss function is crucial for optimizing neural networks and significantly enhancing organ segmentation precision. This area of research remains essential and continues to be a critical focus for further advancements.

### Weakly supervised methods

Obtaining simultaneous annotations for multiple organs on the same medical image poses a significant challenge in image segmentation. Existing datasets, such as LiTS [213], KiTS (p19) [214], and pancreas datasets [215], typically provide annotations for a single organ. How to utilize these partially annotated datasets to achieve a multi-organ segmentation model has arisen increasing interest.

Early methods involved training a segmentation model for each partially annotated dataset, and then combining the output of each model to obtain multi-organ segmentation results, referred to as multiple networks. Although this method is intuitive, it increases computational complexity and storage space. Later, Chen et al*.* [216] improved upon the multiple networks method by introducing a multi-head network. This network consists of a task-shared encoder and multiple task-specific decoders. When an image with annotations for a specific organ is input into the network, only the decoder parameters corresponding to that organ are updated, while the parameters for decoders corresponding to other organs are frozen. Though the multi-head network represents an improvement over multiple networks, this architecture is not flexible and cannot easily adapt to a newly annotated dataset. Recently, various methods have been proposed to use these partially annotated datasets, primarily falling into two categories: conditional network-based methods and pseudo-label-based methods.

### Conditional network-based methods

**Fig. 9:**
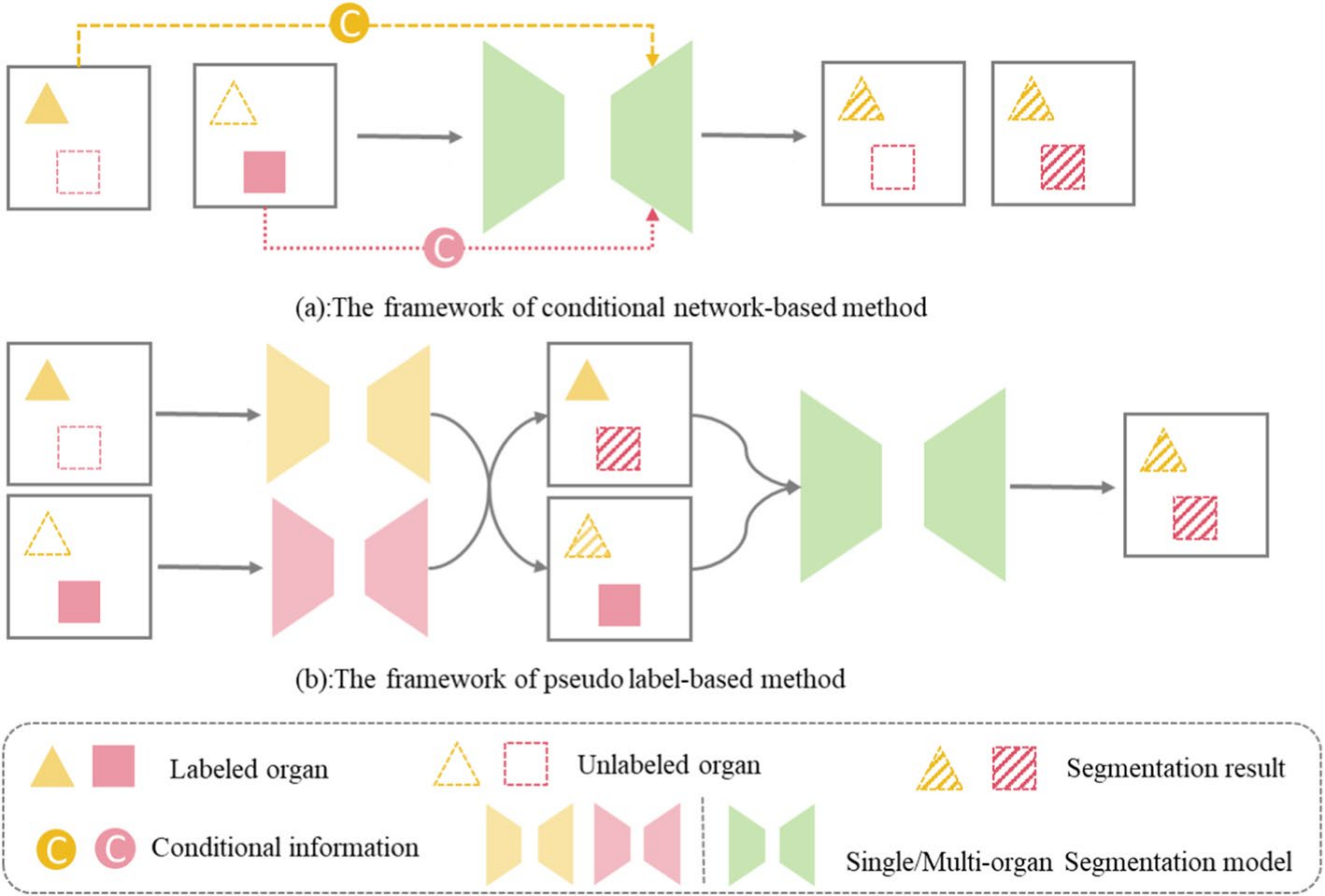
Framework of partially annotated-based-methods

Conditional network-based methods primarily involve embedding conditional information into the segmentation network, thus establishing a relationship between parameters of the segmentation model and the target segmented organs, which is shown in Fig. 9(a). Considering the way in which conditional information is incorporated into the segmentation network, methods based on conditional networks can be further categorized into task-agnostic and task-specific methods. Task-agnostic methods refer to cases where task information and the feature extraction by the encoder–decoder are independent. Task information is combined with the features extracted by the encoder and subsequently converted into conditional parameters introduced into the final layers of the decoder. Typical methods include DoDNet [217] and its variations [218], which utilized dynamic controllers to generate distinct weights for different tasks, and these weights were then incorporated into the final decoder layers to facilitate the segmentation of various organs and tumors.

Task-specific methods involve incorporating task information into the process of segmentation feature extraction by the encoder–decoder. For example, Dmitriev et al. [219] encoded task-related information into the activation layer between convolutional layers and nonlinear layers of decoder. Tgnet [220] adopted a task-guided method to design new residual blocks and attention modules for fusing image features with task-specific encoding. CCQ [221] embedded class relationships among multiple organs or tumors and utilizes learnable query vectors representing semantic concepts of different organs, achieving new state-of-the-art results on large partially annotated MOTS dataset.

However, currently, most methods based on conditional networks encode task information as one-hot labels, neglecting the prior relationships among different organs and tumors. Recently, foundation models [33] have seen significant development. Contrastive Language-Image Pretraining (CLIP) [222] can reveal the inherent semantics of anatomical structures by mapping similar concepts closer together in the embedding space. Liu et al*.* [223] was among the pioneers in applying CLIP to medical imaging. They introduced a CLIP-driven universal model for abdominal organ segmentation and tumor detection. This model achieved outstanding segmentation results for 25 organs based on 3D CT images and demonstrated advanced performance in detecting six types of abdominal tumors. The model ranked first on the MSD public leaderboard [41] and achieved state-of-the-art results on BTCV dataset [34]. However, since CLIP is predominantly trained on natural images, its capacity for generalization on medical images is constrained. Ye et al*.* [224] introduced a prompt-driven method that transformed organ category information into learnable vectors. While prompt-based methods could capture the intrinsic relationships between different organs, randomly initialized prompts may not fully encapsulate the information about a specific organ.

### Pseudo-label-based methods

Pseudo-label-based methods initially train a segmentation model on each partially annotated dataset. Then, they utilize the trained models to generate pseudo labels for corresponding organs on other datasets, resulting in a fully annotated dataset with pseudo labels. A multi-organ segmentation model is subsequently trained using this dataset, which is shown in Fig. 9(b). Clearly, the performance of the final multi-organ segmentation model is closely tied to the quality of the generated pseudo-labels. In recent years, numerous methods have been proposed to enhance the quality of these pseudo-labels. Huang et al*.* [225] proposed a weight-averaging joint training framework that can correct the noise in the pseudo labels to train a more robust model. Zhang et al*.* [226] proposed a multi-teacher knowledge distillation framework, which utilizes pseudo labels predicted by teacher models trained on partially labeled datasets to train a student model for multi-organ segmentation. Lian et al*.* [176] improved pseudo-label quality by incorporating anatomical priors for single and multiple organs when training both single-organ and multi-organ segmentation models. For the first time, this method considered the domain gaps between partially annotated datasets and multi-organ annotated datasets. Liu et al*.* [227] introduced a novel training framework called COSST, which effectively and efficiently combined comprehensive supervision signals with self-training. To mitigate the impact of pseudo labels, they assessed the reliability of pseudo labels through outlier detection in latent space and excluded the least reliable pseudo labels in each self-training iteration.

### Other methods

The issue of partially annotated can also be considered from the perspective of continual learning. Continual learning primarily addresses the problem of non-forgetting, where a model trained in a previous stage can segment several organs. After training, only the well-trained segmentation model is retained, and the segmentation labels and data become invisible. Next state, when new annotated organs become available, the challenge is how to ensure that the current model can both segment the current organs and not forget how to segment the previous organs. Inspired by [228], Liu et al*.* [229] first applied continual learning to aggregate partially annotated datasets in stages, which solved the problem of catastrophic forgetting and the background shift. Xu and Yan [230] proposed Federated Multi-Encoding U-Net (Fed-MENU), a new method that effectively uses independent datasets with different annotated labels to train a unified model for multi-organ segmentation. The model outperformed any model trained on a single dataset or on all datasets combined. Zhang et al*.* [231] proposed an innovative architecture specifically for continuous organ and tumor segmentation, in which a lightweight, class specific head was used to replace the traditional output layer, thereby improving flexibility in adapting to emerging classes. At the same time, CLIP was embedded into the heads of specific organs, which encapsulates the semantic information of each class through extensive image text collaborative training, this information would be an advantage for training new classes with pre-known class names. Ji et al*.*[232] introduced a novel CSS framework for the continual segmentation of a total of 143 whole-body organs from four partially labeled datasets. Utilizing a trained and frozen General Encoder alongside continually added and architecturally optimized decoders, this model prevents catastrophic forgetting while accurately segmenting new organs.

Others solved this problem from alternative perspectives. Zhou et al*.* [175] proposed a Prior-aware Neural Network (PaNN) that guided the training process based on partially annotated datasets by utilizing prior statistics obtained from a fully labeled dataset. Fang and Yan [233] and Shi et al*.* [234] trained uniform models on partially labeled datasets by designing new networks and proposing specific loss functions.

In multi-organ segmentation tasks, weak annotation not only includes partial annotation, but also includes other forms such as image-level annotation, sparse annotation, and noisy annotation [235]. For example, Kanavati et al*.* [236] proposed a weakly supervised method for the segmentation of liver, spleen, and kidney based on classification forests, where the organs were labeled through scribbles.

### Semi-supervised methods

Semi-supervised methods are gaining popularity in organ segmentation due to their ability to enhance segmentation performance while reducing the annotation burden. These methods have found application in diverse medical image segmentation tasks, such as heart segmentation [237–239], pancreas segmentation [240], and tumor target region segmentation [241]. In a comprehensive review by Jiao et al*.* [242], the authors categorized semi-supervised learning methods in medical image segmentation into three paradigms: pseudo-label-based, consistency regularization-based, and knowledge prior-based methods. In this work, we specifically focus on exploring semi-supervised methods for multi-organ segmentation.

Ma et al*.* [39] proposed a semi-supervised method for abdominal multi-organ segmentation using pseudo-labeling. Initially, a teacher model was trained on labeled datasets to generate pseudo labels for unlabeled datasets. Subsequently, a student model was trained on both the labeled and pseudo-labeled datasets, and the student model replaced the teacher model for final training.

Semi-supervised multi-organ segmentation often employs multi-view methods to leverage information from multiple image planes and improve the reliability of pseudo-labels. Zhou et al*.* [243] proposed the DMPCT framework, which incorporated a multi-planar fusion module to iteratively update pseudo-labels for different configurations of unlabeled datasets in abdominal CT images. Xia et al*.* [244] proposed the uncertainty-aware multi-view collaborative training (UMCT) method, which employed spatial transformations to create diverse perspectives for training independent deep networks. Subsequently, these networks were collectively trained using multi-view consistency on unlabeled data, resulting in improved segmentation effectiveness.

Apart from collaborative training, consistency-based learning is another effective approach for multi-organ segmentation, given the diverse organ categories and their dense distribution. This method promotes the consistency of network outputs by using different parameters. For example, Lai et al*.* [245] proposed a semi-supervised DLUNet, which consisted of two lightweight U-Nets in the training phase. Additionally, for unlabeled data, the outputs from both networks were used to supervise each other, improving the segmentation accuracy of these unlabeled data. This method achieved an average DSC of 0.8718 for 13 organs in the abdomen. Chen et al*.* [246] proposed a novel teacher–student semi-supervised multi-organ segmentation model, called MagicNet, which normalized consistency training between teacher and student models by enhancing unlabeled data. MagicNet mainly included two data enhancement strategies, encouraging unlabeled images to learn relative organ semantics (cross-branch) from images and enhancing the segmentation accuracy of small organs (within-branch), Numerous experiments conducted on two common CT multi-organ datasets have demonstrated the effectiveness of MagicNet and were significantly superior to state-of-the-art semi-supervised medical image segmentation methods.

Furthermore, several other methods have been proposed for semi-supervised based method. For example, Lee et al*.* [247] developed a method that employed a discriminator module, which incorporated human-in-the-loop quality assurance (QA) to supervise the learning of unlabelled data. The QA scores were used as a loss function for the unlabelled data. Raju et al*.* [248] proposed an effective semi-supervised multi-organ segmentation method, CHASe, for liver and lesion segmentation. CHASe leverages co-training and hetero-modality learning within a co-heterogeneous training framework. This framework can be trained on a small single-phase dataset and can be adapted for label-free multi-center and multi-phase clinical data.

## Discussion

This paper systematically summarizes the methods of multi-organ segmentation-based on deep learning, mainly from the aspects of data and methodology. In terms of data, it provides an overview of existing publicly available datasets and conducts an in-depth analysis of data-related issues. In terms of methodology, existing methods are categorized into fully supervised, weakly supervised, and semi-supervised based approaches. The proposal of these methods holds significant research significance in advancing automatic segmentation of multiple organs. Future research trends can be considered from the following aspects:

### About datasets

Data play a crucial role in enhancing segmentation performance. Even the simplest models can achieve outstanding performance when trained on a high-quality dataset. However, compared to natural images, there is a shortage of publicly available datasets for multi-organ segmentation, and most methods are trained and tested on private datasets [249]. As summarized in the supplementary materials, many methods proposed in the literature are trained and validated on their own private datasets. This poses challenges in validating the model's generalization ability. Therefore, it is necessary to create a multi-center public dataset with a large volume of data, extensive coverage, and strong clinical relevance for multi-organ segmentation. In order to fully utilize abundant unlabeled data, combining weakly supervised and semi-supervised techniques, and leveraging human expertise in iterative labeling loops, federated learning techniques can be employed to jointly train models using data from various sites while ensuring privacy.

### About fully supervised based methods

Based on the four types of segmentation methods of fully supervised method introduced earlier, future research directions can be considered from the following aspects: firstly, design a new network architecture or investigate how to better integrate different network architectures. Recently, an efficient variant of attention mechanism, Mamba [250, 251], has been proposed, surpassing CNN and Transformer in many medical image analysis tasks. Secondly, considering the respective issues of 2D and 3D architectures, designing lightweight 3D networks while maintaining image information and reducing computational burden is a research approach. Additionally, current multi-view methods only aggregate three perspectives at the result level, with the intermediate feature extraction processes being independent of each other. In the future, it can be explored to leverage the intermediate feature extraction processes or incorporate more view information. Thirdly, combining the characteristics of multiple organs, designing novel plug-and-play modules to enhance multi-organ segmentation performance. Finally, due to differences in organ size, shape irregularity, and imaging quality, deep neural networks exhibit inconsistent performance in medical image multi-organ segmentation. Designing loss functions based on the characteristics of different organs to make the network pay more attention to difficult-to-segment organs is an important research direction.

### About weakly supervised based methods

At present, many pioneering works have been proposed to address the issue of partially supervised based method, but current works mainly consider that each dataset only annotates one organ and only considers CT images. However, in a more general situation, many publicly available datasets have multiple annotated organs, different datasets may have same organs annotated, and there are also datasets with another modality [227]. The future trend is how to design a more general architecture to handle cases with overlapping organs and different modalities.

### About semi-supervised based methods

In medical science, there is a vast amount of unlabeled datasets, with only a small portion being labeled. However, there is limited discussion on semi-supervised approaches for multi-organ segmentation. However, there are a large number of unlabeled datasets in medicine, with only a small amount of data labeled. Utilizing the latest semi-supervised methods and combining prior information such as organ size and position, to improve the performance of multi-organ segmentation models is an important research direction [252, 253].

### About considering inter-organ correlation

In multi-organ segmentation, a significant challenge is the imbalance in size and categories among different organs. Therefore, designing a model that can simultaneously segment large organs and fine structures is also challenging. To address this issue, researchers have proposed models specifically tailored for small organs, such as those involving localization before segmentation or the fusion of multiscale features for segmentation. In medical image analysis, segmenting structures with similar sizes or possessing prior spatial relationships can help improve segmentation accuracy. For example, Ren et al*.*[156] focused on segmenting small tissues like the optic chiasm and left/right optic nerves. They employed a convolutional neural network (CNN)-based approach with interleaved and cascaded processing to handle various tissues, allowing preliminary segmentation results of one organ to assist in improving the segmentation of other organs and its own segmentation. Qin et al*.*[254] considered the correlation between structures when segmenting the trachea, arteries, and veins, including the proximity of arteries to airways and the similarity in strength between airway walls and vessels. Additionally, some researchers [255] took into account that the spatial relationships between internal structures in medical images are often relatively fixed, such as the spleen always being located at the tail of the pancreas. These prior knowledge can serve as latent variables to transfer knowledge shared across multiple domains, thereby enhancing segmentation accuracy and stability.

### About combining foundation model

Traditional methods involve training models for specific tasks on specific datasets. However, the current trend is to fine-tune pretrained foundation models for specific tasks. In recent years, there has been a surge in the development of foundation model, including the Generative Pre-trained Transformer (GPT) model [256], CLIP [222], and Segmentation Anything Model (SAM) tailored for segmentation tasks [59]. These models have achieved breakthrough results on natural images. However, due to their training samples being mostly natural images with only a small portion of medical images, the generalization ability of these models in medical images is limited [257, 258]. Recently, there have been many ongoing efforts to fine-tune these models to adapt to medical images [58, 257]. For the problem of multi-organ segmentation, it is possible to train a specialized segmentation model for medical images by integrating more medical datasets, or study better fine-tuning methods, as well as integrate knowledge from multiple foundation models to improve the segmentation performance.

## Conclusion

We provide a systematic review of 195 studies on multi-organ segmentation-based on deep learning. It covers two main aspects: datasets and methods, encompassing multiple body regions such as the head, neck, chest, and abdomen. We also propose tailored solutions for some of the current challenges and limitations in this field, highlighting future research directions. Our review indicates that deep learning-based multi-organ segmentation algorithms are rapidly advancing towards a new era of more precise, detailed, and automated analysis.

## Data Availability

Not applicable.
